# Accurate Identification and Analysis of Human mRNA Isoforms Using Deep Long Read Sequencing

**DOI:** 10.1534/g3.112.004812

**Published:** 2013-03-01

**Authors:** Hagen Tilgner, Debasish Raha, Lukas Habegger, Mohammed Mohiuddin, Mark Gerstein, Michael Snyder

**Affiliations:** * Department of Genetics, Stanford University, Stanford, California 94305; † Department of Molecular, Cellular and Developmental Biology, Yale University, New Haven, Connecticut 06120; ‡ Program in Computational Biology and Department of Molecular Biophysics and Biochemistry, Yale University, New Haven, Connecticut 06120; § Roche, Branford, Connecticut 06405

**Keywords:** RNA, Roche sequencing, human, splicing, transcriptome

## Abstract

Precise identification of RNA-coding regions and transcriptomes of eukaryotes is a significant problem in biology. Currently, eukaryote transcriptomes are analyzed using deep short-read sequencing experiments of complementary DNAs. The resulting short-reads are then aligned against a genome and annotated junctions to infer biological meaning. Here we use long-read complementary DNA datasets for the analysis of a eukaryotic transcriptome and generate two large datasets in the human K562 and HeLa S3 cell lines. Both data sets comprised at least 4 million reads and had median read lengths greater than 500 bp. We show that annotation-independent alignments of these reads provide partial gene structures that are very much in-line with annotated gene structures, 15% of which have not been obtained in a previous *de novo* analysis of short reads. For long-noncoding RNAs (*i.e.*, lncRNA) genes, however, we find an increased fraction of novel gene structures among our alignments. Other important aspects of transcriptome analysis, such as the description of cell type-specific splicing, can be performed in an accurate, reliable and completely annotation-free manner, making it ideal for the analysis of transcriptomes of newly sequenced genomes. Furthermore, we demonstrate that long read sequence can be assembled into full-length transcripts with considerable success. Our method is applicable to all long read sequencing technologies.

Although genome sequencing has become commonplace, a significant challenge is to obtain an accurate annotation of each gene locus as well as a quantitative description of the transcript isoforms produced from it. This information is crucial for inferring biological meaning from each gene locus and, given the role of alternative splicing in diseases such as cancer (David and Manley 2010), will likely play a role in analysis of human disease. Currently, gene annotation is usually addressed using reverse-transcription polymerase chain reaction experiments (Harrow *et al.* 2006, 2012; Pruitt *et al.* 2012) and a quantitative description is carried out using short read sequencing projects (Mortazavi *et al.* 2008; Nagalakshmi *et al.* 2008; Wang *et al.* 2009; Wu *et al.* 2010; Djebali *et al.* 2012). Thus, quantitative description often relies on the presence and quality of existing annotations.

In contrast to lower eukaryotes such as yeast, human transcripts contain more introns on average. It has been estimated that between 74% and 100% of human multiexon genes are alternatively spliced (Johnson *et al.* 2003; Harrow *et al.* 2006; Pan *et al.* 2008; Wang *et al.* 2008). These studies demonstrate that alternative splicing can be highly regulated. Therefore, proper interpretation of eukaryotic transcriptomes requires accurate detection and quantification of full transcripts with accurate assignment of transcription start site, poly-adenylation site, and splice junctions. Mapping short reads against a genome annotation (Wang *et al.* 2008; Graveley *et al.* 2011; Djebali *et al.* 2012) and its junctions provides quantifications of single junctions and exons. Yet prediction of entire transcript structures requires advanced analysis tools (Montgomery *et al.* 2010; Roberts *et al.* 2011). Despite recent advances in the field, it is not always clear how well they reconstruct full-length transcripts when used *de novo*.

Here, we sequence millions of complementary DNAs (cDNAs) in the human K562 and HeLa cell lines using the 454/Roche long-read sequencing technology. Although the reads are shorter than what can be achieved on the Pacific Biosciences platform (Eid *et al.* 2009), the presented data are considerably deeper than what can be achieved at this point on that platform at a reasonable cost. These 454 reads usually span multiple introns and therefore show a level of information that is not present in short read sequencing projects. We show that the partial, but long, gene structures provided by alignments of 454 reads correspond well with annotated transcript structures. In addition, we detected novel, unannotated spliceforms. These results show both the high quality of the annotation and of our sequenced cDNAs, and that the process of genome annotation and transcriptome quantification can be performed without *a priori* knowledge of transcript structures. Similarly, we show that high-quality cell type/condition/individual-specific splicing analysis can be achieved using this approach. The demonstrated approach is in principle applicable to all long-read sequencing approaches, including Pacific Biosciences (Eid *et al.* 2009), and its success will increase with sequencing depth and read length. Overall, this approach is ideal for transcriptome analysis in recently sequenced genomes that lack a detailed annotation or in cases where using an annotation could introduce a bias into the results.

## Materials and Methods

### Data access

**Figure 1:**
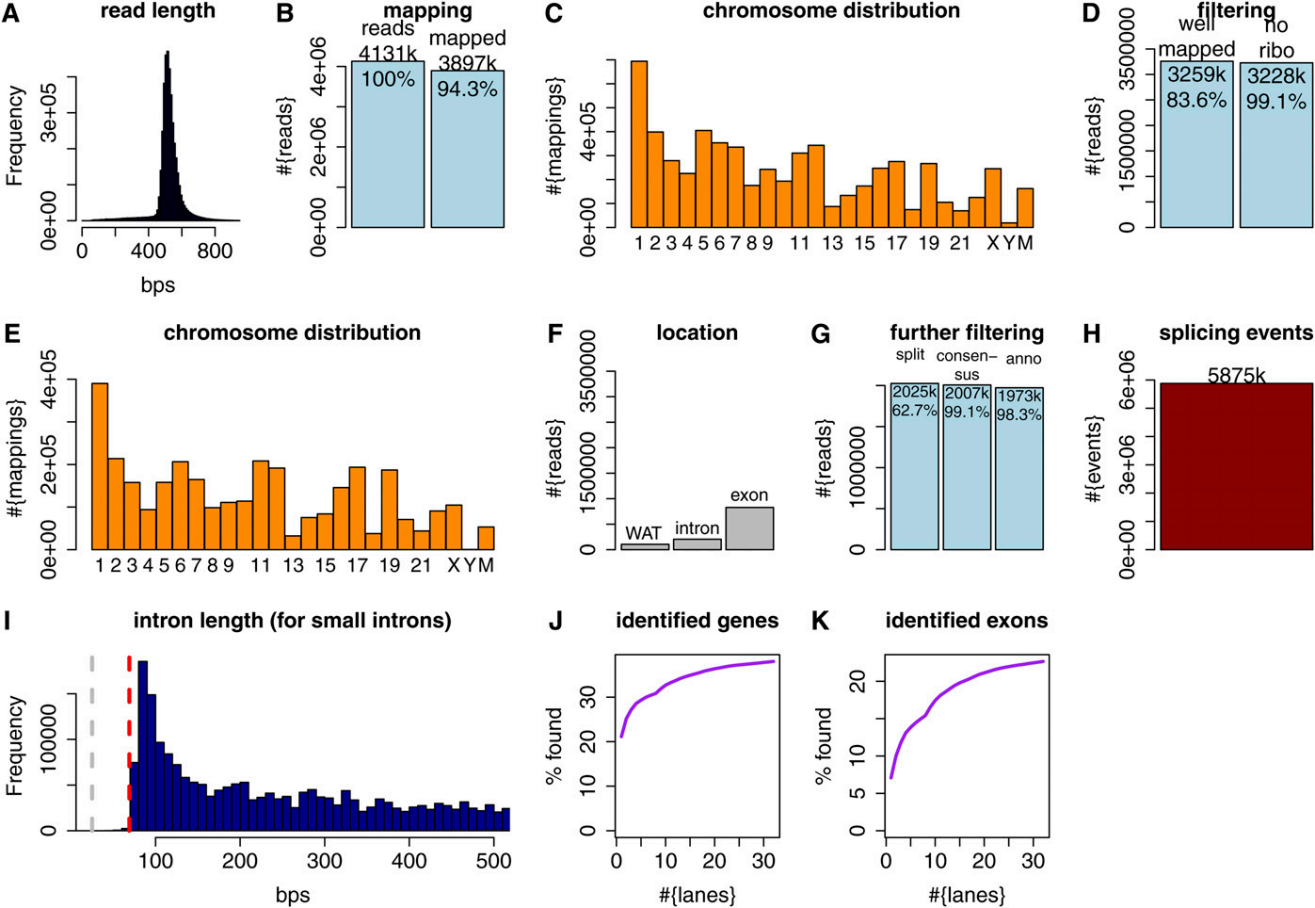
(A) Read length histogram for the K562 cell-line. (B) Total number of reads in the K562 cell-line and number (and percentage) of reads that could be mapped using GMAP. Percentages in light blue bars are given with respect to the previous light blue bar. (C) Chromosome distribution of read-mappings. (D) Number of reads (and percentage) that were considered mapped with high confidence (“well-mapped”) and number of reads (and percentage) of reads that did not overlap ribosomal RNA genes. (E) Chromosome distribution of high confidence read mappings that did not overlap ribosomal RNA genes. (F) Number of reads falling entirely into regions without annotated transcription (WAT), intronic, and exonic regions. (G) Number and percentage (with respect to the previous light blue bar) of reads containing a split (first bar); number and percentage of reads containing at least one split and having intron-consensus di-nucleotides at the ends of all splits (second bar); number and percentage of reads containing at least one split and having intron-consensus di-nucleotides at the ends of all splits and having at least one split-end as an annotated splice site for all splits (third bar). (H) Number of introns in these reads (with respect to last blue bar in G). (I) Intron length distribution for the previous introns, showing only introns of up to 500 bps. (J) Percentage of annotated genes identified when using increasing number of reads. (K) Percentage of annotated exons identified when using increasing number of reads.

The 454 reads (sff-files) are available at the Sequence Read Archive (SRA) under accession no. SRA063146. These data also are available currently at http://homes.gersteinlab.org/people/lh372/ENCODE_454/K562/ and http://homes.gersteinlab.org/people/lh372/ENCODE_454/HeLaS3/. Well-aligned alignments (used in Figure 1E and Supporting Information, Figure S2E) for K562 and HeLa S3 are available as supplementalFileS1.gff.gz and supplementalFileS2.gff.gz at http://stanford.edu/~htilgner/2012_454paper/454.index.html. Note that for spliced alignments the alignment strand has been changed to RNA-direction (as indicated by the dinucleotide-consensus) and alignments overlapping ribosomal RNA genes have been removed.

### RNA extraction and sequencing

For RNA isolation the cells were grown to 60–70% confluency and lysed using Trizol (Life Technologies). Total RNA was extracted from the lysate following the protocol provided by the vendor (Life Technologies). RNA was digested with DNase, extracted with phenol:chloroform (1:1), and precipitated using ethanol. Ribosomal RNA was removed using RiboMinus technology (Life Technologies), and mRNA was purified using oligo-dT columns (Life Technologies). Purified mRNA was provided to 454 for subsequent processing. Roughly speaking, from 200 ng of messenger RNA as starting material, a cDNA library was prepared using Roche kits (Primer Random, cDNA Synthesis System, and the GS Rapid Library Prep Kit), and 32 cells were sequenced for each cell-line.

### RNAseq mapping of long RNAseq reads

Fasta sequence was mapped to the human hg19-genome sequence using the Genomic Mapping and Alignment Program (GMAP) and a minimal intron length of 25 (–minIntronlength = 25).

### Criteria for high-confidence mappings (“well-mapped reads”)

For each read-alignment, we calculated two numbers:

- mapLength: the ratio of the number of aligned bases and the read length; and
- mapQuality: the fraction of identically aligned bases, averaged over all exonic blocks of the read-alignment.

Read-alignments for which mapLength <0.67 or mapQuality <0.75 were discarded. For reads with multiple alignments, the read-alignment A that maximized mapLength* mapQuality was retained if and only if 0.98*mapLength_A_* mapQuality_A_ ≥ mapLength_B_* mapQuality_B_ for all other alignments B of the read.

### Identification of genes for a mapped and spliced read

To each spliced read, we assigned the spliced gene with which it shared most splice sites. If two genes fulfilled this criterion, both were retained.

### Inclusion and exclusion reads for mapped internal exons

For each cell type *t* and all exons *ex* that appeared as internal in at least one read, we determined two numbers:

1. IR_t_(ex): the number of reads linking a splice site of this exon to an upstream exon and/or a downstream exon.
2. ER_t_(ex): the number of reads linking an upstream exon of *ex* to a downstream exon of *ex*, with at least 50nts separating ex and both neighboring exons

### Minimal evidence for alternative splicing

Each internal exon ex of a read mapping was considered for testing of AS if and only if the following conditions were met:

- the number of reads (in both cell types combined), in which ex appeared as an internal exon was at least 2;
- we found at least one read, indicating the usage of at least one of the splice sites in one cell type, and at least one read skipping this exon in the other cell type (or vice-versa); and
- the sum of IR_K562_(ex), ER_K562_(ex), IR_HelaS3_(ex), ER_HelaS3_(ex) was greater than (or equal to) 0.75 times the number of reads overlapping the exon ex.

### Inclusion level (or Ψ-value) definition

For each identified AS-exon we defined the Ψ-value in cell type t as the ratio of IR_t_(ex) and (IR_t_(ex) + ER_t_(ex)).

### Splice-site strength measure

For each exon we used geneid (Parra *et al.* 2000) to calculate an acceptor score and a donor score. Non-AG acceptors were assigned a score of −15 and non-GT donors a score of −7.

### CDS coding exons

Exons, which were entirely coding in at least one annotated transcript, were labeled “CDS.” Importantly, this does not exclude the possibility that they might be partially or entirely noncoding /UTR in other transcripts.

### Alternatively skipped exon calling

For exons with “minimal evidence for AS” (see the section *Minimal evidence for alternative splicing*), we constructed a 2 × 2 table, containing the numbers IR_K562_(ex), ER_K562_(ex), IR_HelaS3_(ex), ER_HelaS3_(ex). All resulting tables were subjected to two one-sided Fisher tests, and a Benjamini-Hochberg correction was applied to both directions.

### Using cufflinks to predict full-length transcript structures based on 454 reads

We discarded 454 reads for which any aligned block (exon) had a GMAP score of less than 98 (*i.e.*, <98% of the aligned base-pairs are matches). The remaining reads were submitted to cufflinks using a minimal intron length of 25bps (-min-intron-length 25).

### ENCODE predicted full-length transcripts (based on Cufflinks and ENCODE short read RNAseq)

As part of the ENCODE project, the previously cited Djebali and coworkers have produced large amounts of paired-end 76-bp RNAseq data. Among other things, these authors have predicted transcript models in different subcellular compartments and cell types using Cufflinks. We downloaded the full-length predictions corresponding to K562, whole-cell, long, and polyadenylated RNA-seq from http://hgdownload-test.cse.ucsc.edu/goldenPath/hg19/encodeDCC/wgEncodeCshlLongRnaSeq/releaseLatest/wgEncodeCshlLongRnaSeqK562CellPapTranscriptDeNovo.gtf.gz. These transcript models sometimes contained very short introns (*e.g.*, five base-pairs and less). For equivalence with the GMAP alignments (and not to overstate the performance of our 454 approach), very close exon-pairs (separated by less than 25 bp) were joined into one exon.

More information about the original dataset we downloaded can be found at http://genome.crg.es/~jlagarde/encode_RNA_dashboard/hg19_RNA_dashboard_files.txt; the geoSampleAccession is given as GSM765405.

### Human genome sequence

The human genome sequence(Lander *et al.* 2001; Venter *et al.* 2001) version hg19 was downloaded from the UCSC browser (Kent *et al.* 2002) on August 21, 2011.

### Comparison of intron structure

We first denoted each intron by its genomic position (chr_start_end_strand). Each 454 read was then represented by a tuple X = (x_1_,x_2_,…,x_n_) where each x_i_ is an intron. Note that this representation of a read represents perfectly the introns (splits) of a read but loses the information of the start and end of the read. In the same way, we represented each annotated transcript as a tuple Y = (y_1_,y_2_,…,y_m_). We say that tuple X is contained in Y, if and only if there is a k ≥ 1, so that x_1_ = y_k_, x_2_ = y_k+1_,…, x_n_ = y_k+n-1_. In other words the intron structure X (given by a 454 read) is a substructure of the intron structure Y (given by an annotated transcript).

## Results

### Transcriptome sequencing and annotation-free mapping in two human cell lines

Whole-cell cDNA libraries from messenger RNA molecules (*Materials and Methods*) were prepared in two human cell lines: the myelogenous leukemia cell line K562 and cervical cancer HeLa cells. These cDNA libraries were sequenced on the 454-platform using multiple runs, yielding a total number of 4.1 million reads with a median length of 521 bp for K562 cells and 4.6 million reads with a median length of 538 bp for HeLa cells. Read length rarely ever fell below 450 bp, whereas reads with lengths of longer than 800 bp could readily be observed (Figure 1A, supporting information, Figure S1, A and B, and Figure S2A).

All reads were mapped to the hg19 version of the human genome sequence (Lander *et al.* 2001; Venter *et al.* 2001) using the spliced aligner GMAP (Wu and Watanabe 2005). For K562 cells, 94.3% of all reads could be mapped (Figure 1B). The remaining 5.7% unmapped reads were on average of much lower quality and also longer than the mapped reads. A more detailed analysis of unmapped reads can be found in Figure S3 and its legend. Mapping numbers per chromosome broadly correlated with chromosome length (Figure 1C). Chromosome Y received a very low number of read mappings (~19,000). These mappings are unlikely to reflect the true biological origin of the corresponding RNAs but rather homology between DNA sequences on chrY and other chromosomes, because K562 cells were created from a female donor. In contrast to short-read technologies such as Illumina, long-read technologies such as 454 usually obtain reads of varying length. The typical approach of fixing a number of mismatches, above which a mapping for a read is not considered, is therefore not suitable for long-read technologies.

Thus, we devised more flexible criteria (*Materials and Methods*) to define high-confidence mappings (or “well-mapped reads”), aimed at ensuring that the read mapping (1) consisted of a large number of identically matched nucleotides, (2) represented a large fraction of the read and (3) that any secondary mapping for the same read was clearly worse than the read mapping in question. More precisely, we only considered mappings for which at least three quarters of the aligned nucleotide-pairs were identical and the mapping length was at least two thirds of the read length. These criteria were very stringent to guarantee correctness, rather than completeness. A total of 83% (3.25 million) of the mapped reads were considered high confidence (see *Materials and Methods*) and did not overlap ribosomal RNA genes. Thus, 17% the reads’ mappings were removed by our filter (Figure 1D). On chromosome Y, however, 33 of 34 reads were removed by our filter (Figure 1E). This shows that our filter achieves what it intended to achieve, that is, to remove almost all mappings that we know to be false (*e.g.*, mappings to chromosome Y in a female cell-line) while removing few other mappings.

Using annotation projections (A. Tanzer and R. Guigó, unpublished data) defining regions without annotated transcription (WAT) [according to the gencode v7 annotation; these regions contain no protein coding genes, no long-noncoding RNAs (lncRNAs), no small RNA genes, *etc*.], exonic, and intronic regions, we assessed how many mappings fell entirely within one of these nonoverlapping categories. Very few fell entirely within regions without annotated transcription (3.3%) or single intronic regions (6.3%). Hence, at least 9.6% (~310k) of the reads originate from transcription units that are not yet appreciated. A considerable portion (25.6%) fell entirely within single exonic regions and presumably most often represent single exon genes (Figure 1F). The remaining reads overlap regions of at least two different categories. Slightly more than two million reads were split-mappings and for almost all of these we found all split points to respect the intron consensus (GT-AG, GC-AG, or AT-AC) and to use at least one annotated splice site (Figure 1G). We conclude that in nearly all cases, it is possible to determine the location from which an RNA was transcribed as well as the precise location of all its introns, using nothing but the program GMAP and the genome. This entire collection of alignments contained a total of 5.9 million introns (Figure 1H). Annotated human introns are very infrequently shorter than 70 bps, but 70−80 bps introns are frequently observed (Figure S4), suggesting that this is a minimum length for introns to be appropriately processed. The distribution of intron lengths in our 454-mappings is highly consistent with the length-distribution of annotated introns(Figure 1I).

We next monitored how many annotated spliced genes were covered by at least one read, limiting our analysis to genes and reads that contain at least one splicing event. Using only one lane (~90,000 reads), we found that approximately 20% of such genes were covered (defined by splice site identity; see *Materials and Methods*) by at least one read, and this percentage increased gradually to 38% when we used more and finally all 32 lanes (Figure 1J). The coverage percentage began to approach a plateau after about 25 lanes, although saturation was not attained, even after 32 lanes. A similar analysis on exon level showed that about 23% of annotated exons could be found in at least one 454 read (requiring both splice sites to be precisely identified, Figure 1K).

For mappings in HeLa (Figure S2), we generally observed similar trends, although both the numbers of mapped and highly confidently mapped reads were considerably lower. As in K562, unmapped reads tended to be longer and of lower quality (Figure S5, A and B; see also Figure S3 for an explanation in the K562 case), suggesting again that some longer reads contain very low-quality nucleotides. Consistent with this interpretation, we found a longer read-length median and a lower read quality in HeLa (than in K562, Figure S5, C and D), coinciding with a lower number of mapped reads in this cell type.

### Comparison of multi-intron arrangements against annotated transcripts

We next assessed whether our read-mappings gave evidence for transcript structures that were not included in the annotation. To this end we transformed each transcript of the annotation into an ordered tuple of introns and then proceeded similarly for our read-mappings. We then asked how often the entire intron-structure (tuple) of one of our reads was contained in the intron-structure (tuple) of an annotated transcript, starting with the K562 cell-line (*Materials and Methods*). In other words, we asked whether an annotated transcript contained all the splice sites indicated by a 454-alignment, and no other splice site in between these splice sites. Note that for this analysis we used only the reads for which each intron respected the splice-site consensus without considering the annotation during the mapping in any way.

**Figure 2:**
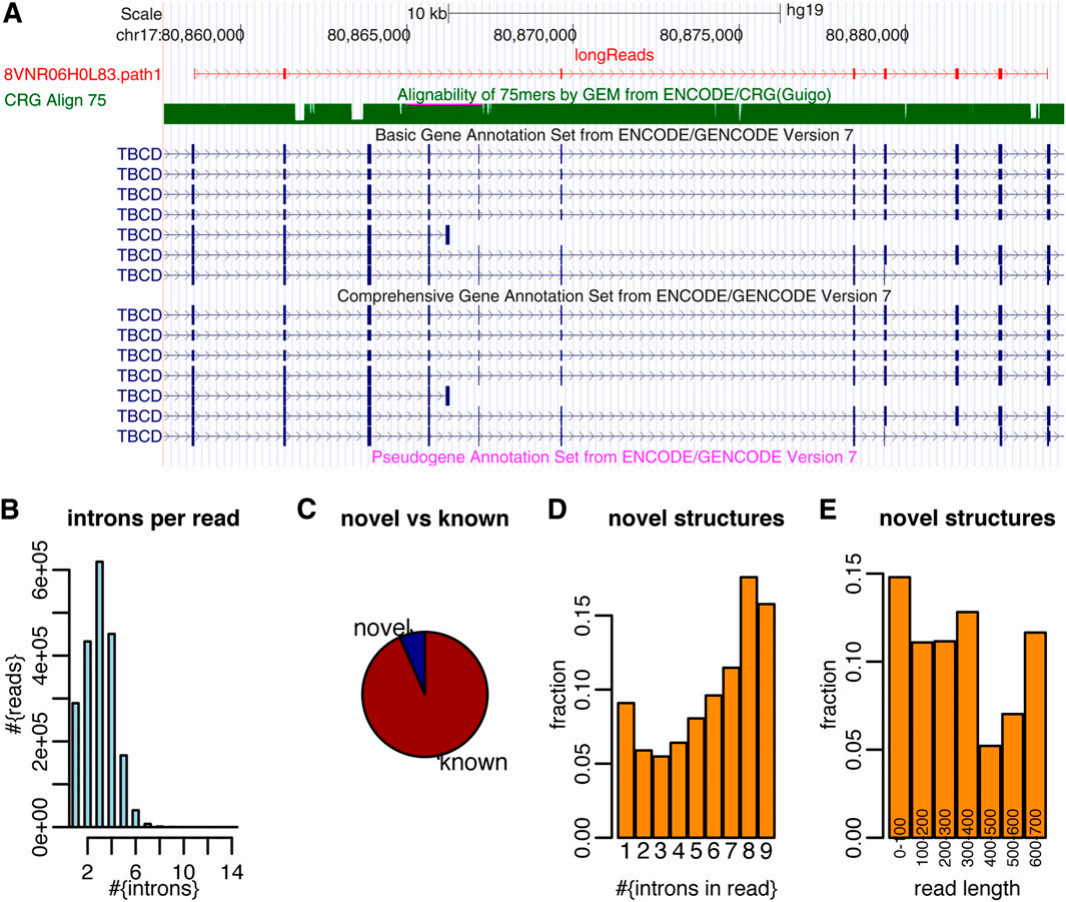
454-read mappings in the K562 cell-line. (A) Example of a 454 read showing a partial gene structure that was not annotated. (B) Distribution of intron number in aligned reads (with consensus splits). (C) Pie chart of partial gene structures (given by alignments of 454 reads) that (1) correspond to parts of annotated gene structures and (2) those that do not correspond to parts of annotated gene structures. (D) Fraction of reads whose intron structures are not included in annotated gene structures as a function of intron number in the read-alignments. (E) Fraction of reads whose intron structures are not included in annotated gene structures as a function of read-length. Note that there are very few reads that have between 0 and 400 bp.

An example of such a novel mapping, in which three exons are simultaneously skipped, is shown in Figure 2A. The ~2 million read-mappings we used in this analysis had at least one intron and more than half a million reads had four introns or more (Figure 2B). Very few (<13,000, 0.6%) had an aligned block with an alignment quality of less than 75%, which might indicate that the K562 genome might not be well represented in hg19 in these regions or that some details of these alignments might be questioned. The vast majority (~93%) of these 454-intron-structures corresponded to partial intron-structures of the annotation (that is, the annotation contains a transcript, that contains all the introns that the read contained and no other introns in between, Figure 2C). This result highlights three facts: First, the high quality of the intron−exon structure in the annotation; second, the high quality of the intron−exon structure in our 454-alignments; and third, although we have used the annotation here to check our results, we would not have needed to do so because our annotation-independent mapping approach finds, in large parts, the same gene structures. The fraction of read-mappings that contained novel elements with respect to the annotation increased quite strongly with the number of introns in the mapped reads and with length of the read from 400 bp on (Figure 2, D and E). Our results also indicate that there appear to be a class of longer gene structures, for which not all transcript structures are described in the annotation. 7% of reads (~133,000 reads) had intron structures that were not fully consistent with annotated intron structures. Only very few fell entirely into intronic or intergenic space (<3000 reads for each of these two categories) and supposedly represent novel genes. Of the aforementioned 133,000 aligned reads, 85% (~113,000) however, had at least one splice site in common with a spliced gene and supposedly represented novel isoforms (or in some cases partially processed transcripts) of these genes. These novel-isoform reads affected 9902 spliced genes and for 4961 spliced reads we could find at least six such reads (data not shown). This finding shows that the vast majority of our reads represent RNA products of known genes, although sometimes with unknown intron structure. In summary, these results show that sequencing long reads is of great use in the annotation of genomes. The simplicity of the RNAseq process in comparison to cloning techniques is a further advantage in this respect. Largely similar results (using HeLa cells; Figure S6) show that transcript reconstruction can be achieved for multiple cells types using this approach.

### Implications for long-noncoding-RNA gene structure

**Figure 3:**
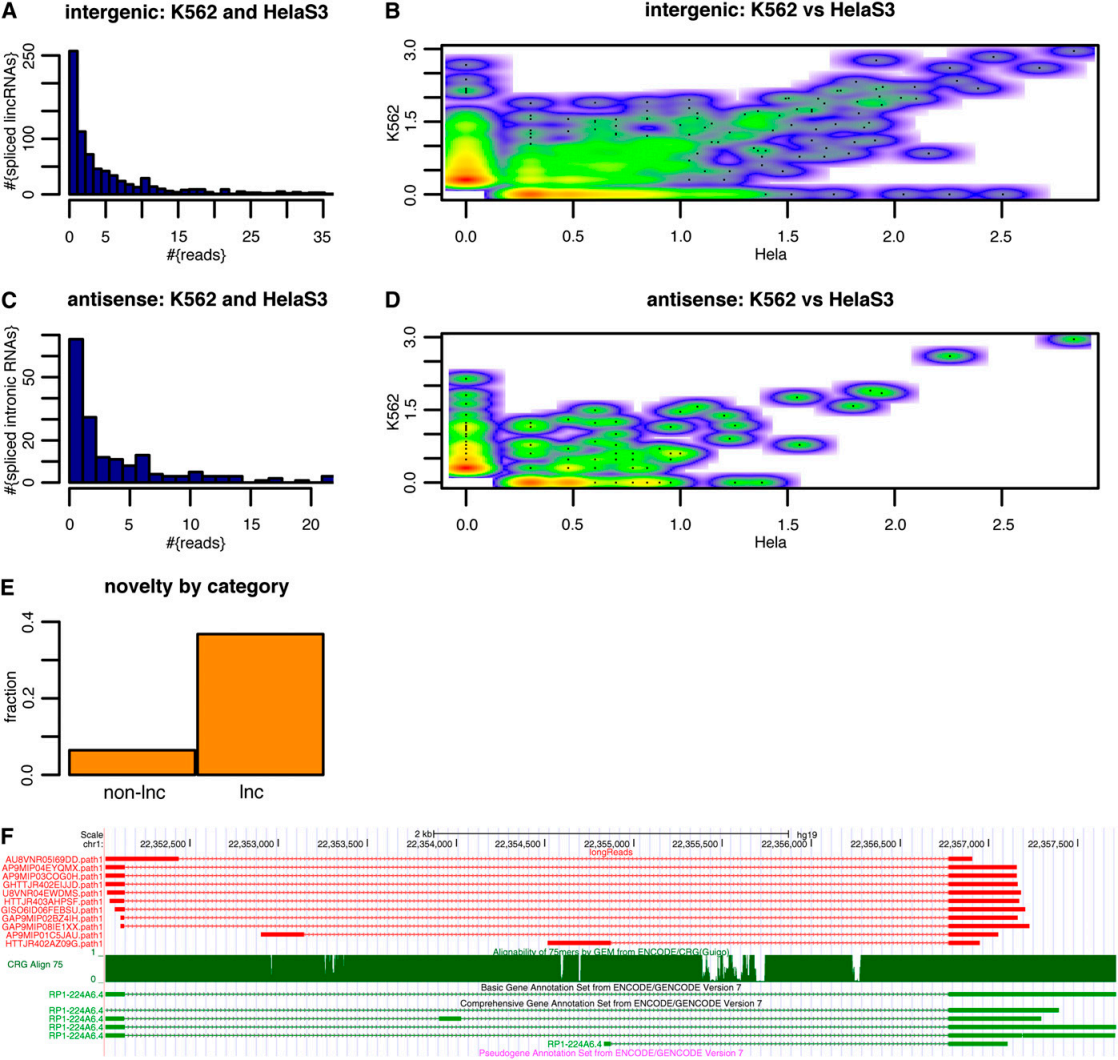
(A) Histogram of long-read numbers found for intergenic, defined as “between protein coding genes” (see Derrien *et al*. 2012), lncRNAs combining the reads found in K562 and in HeLa S3. (B) Scatterplot for log10-transformed read numbers in HeLa S3 (x-axis) and K562 (y-axis) of these lncRNAs. Colorscale from white (no lncRNAs) to red (large number of lncRNAs) (C) Same as A but for intronic lncRNAs. (D) Same as for B but for intronic lncRNAs. (E) Fraction of reads mapping to non-lnc genes that represent novel isoforms (left) and fraction of reads mapping to lncRNA genes that represent novel isoforms (right). (F) First (and not cherry-picked) example of 454 reads (top, red) showing novel isoforms for a lncRNA gene (annotated transcripts in green, bottom). The read that led to the choice of this example is the second to the last.

A class of genes that has received considerable attention in recent years are those that produce fairly long mature RNA products yet do not encode proteins: lncRNAs. The gencode v7 annotation already contains many thousands of such lncRNA genes, so that along with protein coding genes and small noncoding-RNA genes, the total number of genes in this annotation is approximately 50,000. The properties of long-noncoding RNAs in this annotation have recently been described in much detail (Derrien *et al.* 2012). These authors provided a classification of “intergenic” and “intronic” (both with respect to protein coding genes) and also showed that lncRNA genes are on average shorter than protein coding genes and often consist of few exons only. It follows that long-read technologies, such as the 454 reads we employ here, are often able to capture the entire exon−intron structure of such lncRNA genes. We therefore investigated how many of our reads described lncRNA genes. Despite the low and often nuclear expression of lncRNAs (Derrien *et al.* 2012), we could find spliced reads that mapped to 842 spliced intergenic lncRNAs (of 5774 defined by Derrien and coworkers; Figure 3A) and for many of these we could find multiple reads. Some of these were exclusively expressed in either in K562 or in HeLa S3 (Figure 3B). Similarly we could find reads for 193 intronic lncRNA genes and again some were expressed cell type specifically (Figure 3, C and D). Although protein-coding genes have been a topic of research for decades, lncRNA genes have captured attention only much more recently. We therefore investigated how many unknown isoforms our 454-reads could uncover for genes that encode lncRNAs (lnc) and for those that don’t (non-lnc), the latter group being mainly protein coding genes. Although for non-lnc genes, only 6% of all reads supported novel isoforms (Figure 3E, left bar), we found 37% of all reads mapping to lncRNAs to support isoforms that are not known in the gencode v7 annotation. These novel isoforms were given by 4486 spliced reads and affected 599 lncRNA genes. An example of a lncRNA for which novel structures can be found using 454-reads is shown in Figure 3F.

### Comparison of multi-intron arrangements against transcript structures predicted *de novo*

**Figure 4:**
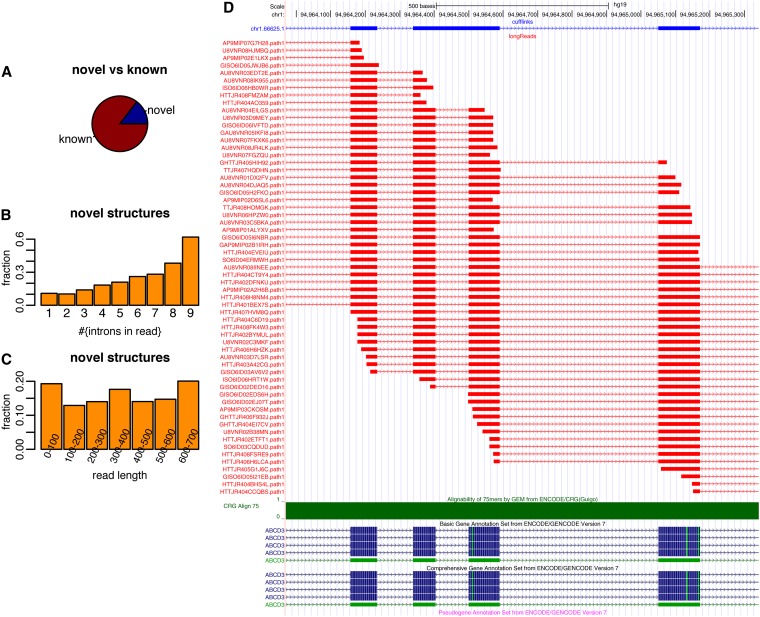
(A) Pie chart (for the K562 cell line) of partial 454 gene structures that (1) correspond to parts of full-length transcript structures predicted using ENCODE short reads and (2) those that do not correspond to parts of these predicted transcript structures. (B) Fraction of reads whose intron-structures are not included in predicted transcript structures (based on ENCODE short reads) as a function of intron number in the read-alignments. (C) Fraction of reads whose intron-structures are not included in predicted transcript structures as a function of read-length. Note that there are very few reads that have between 0 and 400 bp. (D) Example of partial transcript structures given by 454 reads (red) that are not included in predicted cufflinks structures (based on ENCODE short reads from the same cell line, top, blue).

We next assessed whether the partial transcript structures found by mapping long RNAseq reads have been previously obtained through short-read sequencing followed by bioinformatic transcriptome-assembly techniques. To this end we downloaded *de novo* cufflinks-predictions from the ENCODE project (Djebali *et al.* 2012) that are based on short reads (*Materials and Methods*). These predictions were derived from approximately 470 million short reads and often contained exon-pairs separated by few base-pairs. In order to guarantee similar treatment (in comparison with the 454 reads), we joined exon-pairs separated by less than 25 bp within the same transcript. We then compared our 454 alignments against the short-read-cufflinks predictions. More precisely we performed a similar analysis as in Figure 2 using all aligned 454-reads showing at least one intron and for which all introns respected the splice site consensus. The annotation was not considered in this process. Roughly 15% (compared with 7% in Figure 2C) of the intron-structures generated by our alignments of 454 reads were not part of transcript structures predicted within the ENCODE project (Figure 4A). For aligned 454 reads with one, two, or three introns, 90% were found by the ENCODE short-read *de novo* approach; however, this number shifted dramatically when 454-alignments with 7, 8, or 9 introns were considered. For these read-alignments, up to 60% could not be found by the short read *de novo* approach (Figure 4B). This finding highlights that sequencing longer reads will improve our understanding of transcript structures, particularly for genes with larger number of introns. When only considering reads longer than 400 bp (the majority of all 454 reads, see Figure S1A), a greater fraction of novel transcripts was observed for reads between 600 and 700 bps (Figure 4C). Similar results were obtained for HeLa cells (Figure S7). An example of a multi-intron structure that was not correctly identified by the short-read *de novo* approach, but for which 454-alignment and annotation concurred is shown in Figure 4D. Figure S8 shows the next four examples. Two of these involve intron arrangements, for which the short-read cufflinks approach found all parts, but not all existing combinations. Two others are simple alternative splice sites (Figure S8). These results clearly show the need to complement short-read-sequencing with sequencing technologies that can span many introns for accurate transcriptome analysis.

### Analysis of cell type−specific alternative splicing

Since gene expression can vary considerably between cell types, conditions, and individuals, it is crucial to obtain an accurate transcriptome analysis in multiple cell types. Although assessing differences in gene expression is relatively straight forward, analysis of cell type specific alternative splicing often is more complicated. Cell type−specific alternative exons have been determined previously by mapping short reads to exon−exon junctions and collecting for each exon the reads indicating inclusion and exclusion. One can then assess statistically whether the ratio of inclusion reads to exclusion reads is significantly different in two conditions(Wang *et al.* 2008; Graveley *et al.* 2011).

To test whether this approach can be performed in an annotation-independent fashion, we considered all split reads for which the split-point respected the splice site consensus—checks that can be performed in a newly sequenced genome. For all genomic stretches that were indicated to be an internal exon using at least two reads, we collected numbers of three types of reads: (a) reads linking this read to an upstream or downstream exon (or both: “inclusion reads”), (b) reads skipping this exon and 50 adjacent nucleotides on both sides (“exclusion reads”), and (c) the reads overlapping the exon but not falling into category (a) or (b)—see Figure S9 for an example. Whenever category (c) is relatively small in comparison to category (a) and (b), the exon in question has few interfering factors such as alternative acceptors, donors, overlapping transcription start site or poly-adenylation sites that can confuse the analysis. Of these exons, ~47.000 showed minimal evidence of cell type specific splicing (*Materials and Methods*) and were subjected to two one-sided Fisher tests, in which we controlled for multiple testing using the Benjamini-Hochberg method (Benjamini and Hochberg 1995) (false discovery rate ≤ 0.05).

**Figure 5:**
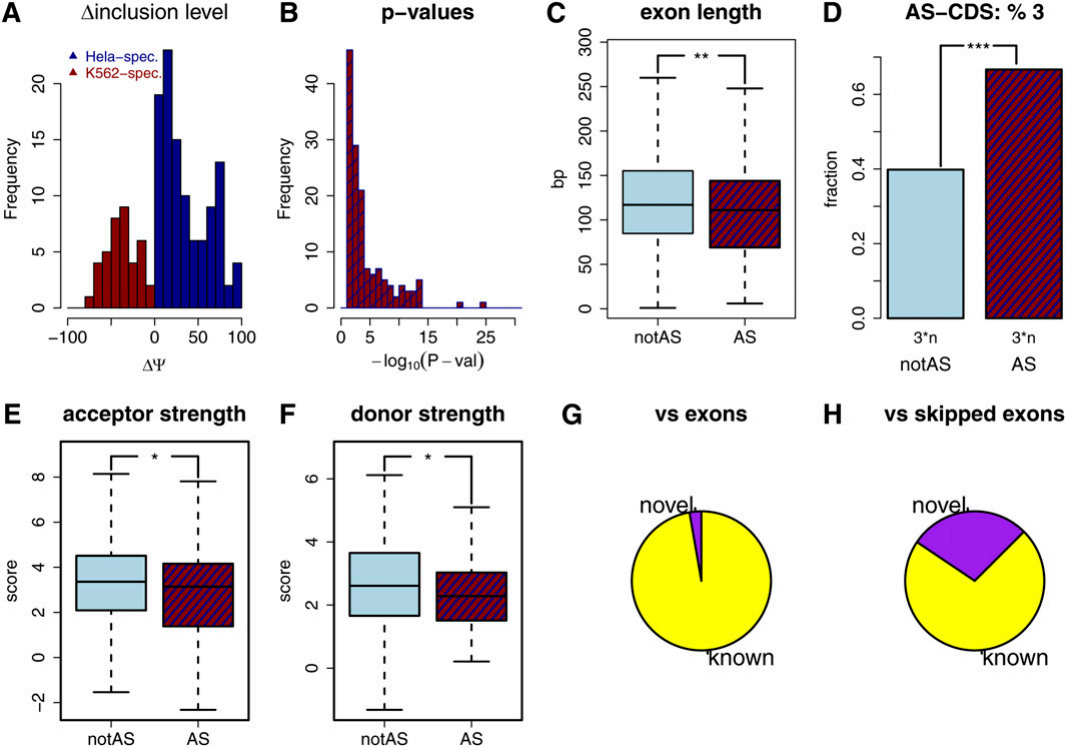
Testing of exons for cell type specific exon inclusion. (A) ΔΨ (*i.e.*, Ψ_HelaS3_ − Ψ_K562_) distribution for exons showing significantly different inclusion levels between the two cell-types. Exons more highly included in the HeLa S3 cell line (blue) and exons more highly included in the K562 cell line (dark red). (B) *P*-value distribution for exons passing the significance threshold of 0.05. (C) Boxplots of exon length for tested exons not passing the significance threshold (light blue) and for those passing the significance threshold (dark blue/dark red). (D) Bar plot indicating the fraction of exons whose length is a multiple of three for tested exons not passing the significance threshold (light blue) and for those passing the significance threshold (dark blue/dark red). (E) Boxplots of acceptor scores for tested exons not passing the significance threshold (light blue) and for those passing the significance threshold (dark blue/dark red). (F) Boxplots of donor scores for tested exons not passing the significance threshold (light blue) and for those passing the significance threshold (dark blue/dark red). (G) Fraction of cell-type specific alternative exons that are annotated as exons (“known”), and those that are not (“novel”). (H) Fraction of cell-type specific alternative exons that are annotated as alternative exons (“known”), and those that are not (“novel”).

A total of 146 exons were found to exhibit cell-type specifically increased inclusion levels. Note that sometimes two or more exons are part of one splicing event, such as double exon-skipping or mutually exclusive exons. The inclusion level or Ψ-value (“percent-spliced-in”) under these circumstances can be defined as the ratio of (a) and (a) + (b) and the definition of the ΔΨ between the two cell types follows directly (*Materials and Methods*). Figure 5A shows the ΔΨ-values for all alternative exons more highly included in HeLa cells and those more highly included in K562 cells. Of these exons, 86% showed a ΔΨ-value equal or higher than a threshold of 10% (corresponding for example to 40% inclusion in one cell type and 50% inclusion in the other, Figure 5A), which has been suggested to guarantee increased biological significance(Wang *et al.* 2008). A total of 40% of the aforementioned alternatively skipped exons showed corrected *p*-values that ranged from 0.05 to 0.005, but the majority had far lower *p*-values (Figure 5B). Because most sequencing technologies have intrinsic biases, it is of utmost importance to independently assess the results and rule out technical artifacts. In the case of alternative splicing, a large body of research has established that alternatively spliced exons exhibit properties that can be readily determined from the genome. Alternatively spliced exons have been shown to exhibit lower splice-site strength and shorter exon length relative to constitutive exons (Zheng *et al.* 2005). In addition, when coding, alternative exons tend to show a length divisible by three (Magen and Ast 2005; Zheng *et al.* 2005), thus keeping the reading frame unchanged when in-/excluded. Consistently, the exons our approach found as alternative showed shorter exon length and increased divisibility of their length by 3 than tested exons that did not pass the significance threshold (Figure 5, C and D). We then calculated acceptor and donor scores for all exons using geneid (Parra *et al.* 2000). As expected, the exons our approach found to be alternatively included between the two cell types showed lower acceptor and donor strength than exons whose inclusion did not differ significantly between the two cell types (Figure 5, E and F). Hence it is possible to search for high quality alternative exons in a completely annotation-free manner using long-read technologies.

Although the annotation was not used to define these alternative exons, practically all of the AS-exons found (97%, 142 of 146) corresponded exactly to known exons (Figure 5G), showing again the high fidelity of this approach. However, approximately 30% of these alternatively included exons were not annotated as alternatively spliced, showing that long read sequencing can be used to improve annotations (Figure 5H).

### Construction of full-length transcripts

**Figure 6:**
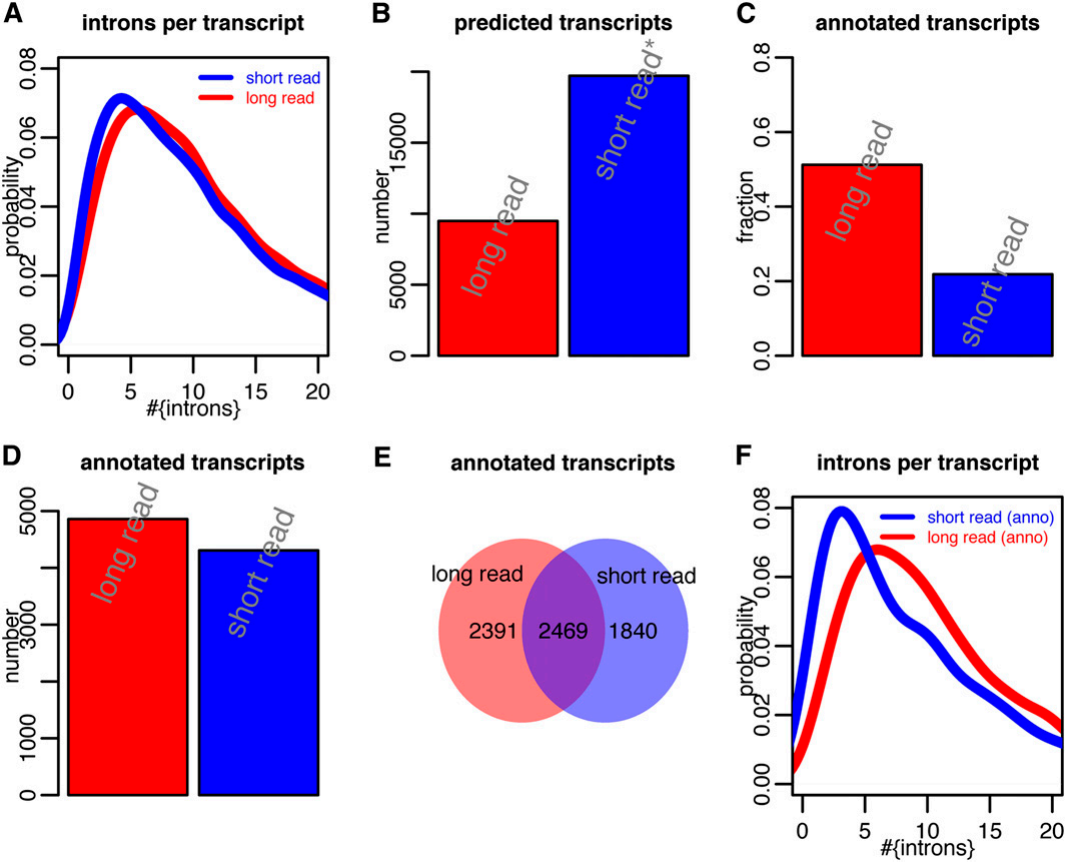
(A) Intron number distribution for short-read-cufflinks transcripts and long-read-cufflinks transcripts. (B) Total number of predicted transcripts for short-read-cufflinks and for long-read-cufflinks. (C) Fraction of predicted transcripts (for short-read-cufflinks and for long-read-cufflinks), for which an annotated transcript with identical introns could be found. (D) Total number of predicted transcripts (for short-read-cufflinks and for long-read-cufflinks), for which an annotated transcript with identical introns could be found. (E) Total number of predicted transcripts with intron-identical annotated transcripts that could be found only by “short-read-cufflinks,” only by “long-read-cufflinks,” or by both. (F) Intron number distribution for intron-identical annotated transcripts that could be found only by “short-read-cufflinks” or only by “long-read-cufflinks.”

The aligned 454 reads represent partial transcript structures sometimes containing double-digit numbers of introns. In the absence of a method to sequence full-length transcripts, it is crucial to combine these partial gene structures into full-length gene structures. We therefore used cufflinks to predict full-length transcripts from our aligned 454 reads (*Materials and Methods*), a task much easier due to the multiple introns that most 454 read-alignments contain. This resulted in a total of 9488 predicted full-length structures. We also downloaded all cufflinks predicted gene structures that were obtained from short read sequencing within the ENCODE project (Djebali *et al.* 2012) (*Materials and Methods*), focusing only on those that overlapped our long-read-cufflinks gene structures. The former set of predicted transcripts (“long-read-cufflinks”) showed significantly more introns than the short-read-cufflinks transcripts, but this difference was not very pronounced (Figure 6A). The absolute number of predicted transcripts was twofold greater in the case of the short-read-cufflinks approach (Figure 6B). For more than 50% of long-read-transcripts, we could find an annotated transcript with identical introns, showing the extraordinary quality of the predicted long-read-cufflinks transcripts. For short-read-cufflinks transcripts, this was only true for 22%, showing that long reads provide a considerable advantage in terms of constructing full-length transcripts (Figure 6C). Note, that in both cases many more transcripts may still have considerably similar intron structures in comparison to annotated transcripts, and the difference may be limited to one or two splice sites. Because of the greater number of predicted transcripts when using short-reads, this however resulted in almost as many transcripts for which an intron-identical annotated transcript existed for the short-read-cufflinks approach as for the long-read approach (Figure 6d). Almost 2500 annotated transcripts were identified by both approaches. However, approximately 2400 annotated transcripts were found only by the long-read approach and almost 1900 only by the short- read approach (Figure 6E). The former showed significantly more introns than the latter, showing that when it comes to assembling transcript structures with high intron numbers, the long-read approach provides a significant advantage over short read sequencing (Figure 6F).

## Discussion

An accurate description of the ensemble of RNA molecules that is encoded by a genome is an integral step toward understanding the biology of the corresponding genome. Usually it takes years until a relatively complete and cell type−specific transcriptome description is made available. Here, we show that using long read sequencing technologies we can provide a very large part of such a description almost instantaneously without further input information.

The accuracy of the provided information is evident by the fact that the vast majority of partial gene structures we find are already part of the gencode v7 annotation, although a large number of novel structures can be found. For lncRNA genes we find a particularly high fraction of novel isoforms, which demonstrates that long-read sequencing can further the understanding of lncRNAs. When comparing our partial gene structures to predicted gene structures based on short read sequencing technologies, however, we find that many of our mappings cannot be reconstructed from short read cDNA sequencing. Hence, sequencing longer reads adds information to short-read analysis of transcriptomes. Nevertheless, one should note that there are many more short-read-predicted transcripts than long-read-predicted transcripts, thus enabling statements about genes, which the long-read approach did not cover.

A clear advantage of short read sequencing is that its low cost allows very deep sequencing, thus enabling quantitative statements about gene or isoform expression and exon inclusion. We therefore explored whether an example of such an analysis, cell type specific exon inclusion, can be performed using long reads. We show that despite the limited sequencing depth and other biases affecting 454 sequencing, such an analysis can be performed in an annotation-independent manner. The thus-defined cell type−specific alternative exons are highly reliable, as 97% of them coincide with already known exons. A total of 30% of these alternative exons were however not alternative according to the annotation; hence, our approach can complete the annotation in a cell type specific manner. The defined alternative exons show characteristics that correspond perfectly to what is known about alternative exons: They show weaker splice sites and are shorter in terms of exon length—and above all, in roughly two-thirds of the cases, they keep the reading frame of the encoded protein.

The dataset we present here comprises millions of reads that span multiple exon-exon junctions. However, it falls short of sequencing entire transcripts. Therefore, the question of whether one can accurately predict full-length transcripts from these reads becomes important. We show that based on our 454 reads we can predict ~9500 full-length transcripts and that for more than half of these an annotated transcript with identical introns exists. This is contrasted by the <25% of all transcripts predicted from short reads that fulfill this criterion. Furthermore, despite the extreme sequencing depth of short reads, more than 2300 annotated full-length transcripts can only be predicted correctly (that is correctness of each single splice site) using 454 reads. Conversely, there are also almost 1900 annotated full-length structures in the regions covered by 454 reads whose exact exon-intron-structures are only found based on short-read-sequencing. This shows, that the length of long-read sequencing and the depth of short-read sequencing complement each other and that their combination can be used to obtain an accurate description of transcriptomes. Importantly, new advances in sequencing technology could help reduce the cost of long read sequencing in comparison to the approach employed here. Coupling isolation of ~450-bp cDNA fragments with 250-bp paired-end MiSeq sequencing could lead to ~450 bp reads and to results that come close to ours. Assuming that Illumina continues to increase read length (from 25 or 36 initially to 150 bp paired-end at the time of writing) this could also be true for that platform as well. Finally, using the Pacific Biosciences platform, we could obtain much longer reads. Due to the lower throughput of this platform, it might be worth to couple this platform with capturing techniques in order to represent all genes independently of their expression level.

In summary, these results show that long-read cDNA sequencing is ideal for transcriptome analysis in species for which an annotation is not available or in cases in which relying on an annotation can introduce biases. In species in which an annotation is available, long-read cDNA analysis can successfully complete the annotation and complement short read sequencing analysis.

## Supplementary Material

Supporting Information
