# Supplementary material for: Accurate Identification and Analysis of Human mRNA Isoforms Using Deep Long Read Sequencing
Source: G3 (Bethesda). 2013 Mar 1;3(3):387–97. doi: 10.1534/g3.112.004812 (PMC3583448; doi:10.1534/g3.112.004812)
Supplement: Supporting Information [file supp_3_3_387__index.html]

Supporting Information 

# Accurate Identification and Analysis of Human mRNA Isoforms Using Deep Long Read Sequencing

## Supporting Information for Tilgner *et al.*, 2013

**Files in this Data Supplement:**

- Supporting Information - Figures S1-S9 (PDF, 4 MB)
- Figure S1 - Read length distribution for reads in the K562 cell-line (a) and in the HelaS3 cell-line (b). (PDF, 86 KB)
- Figure S2 - Read length histogram for the HelaS3 cell-line (a). (PDF, 184 KB)
- Figure S3 - Boxplots for length distribution of mapped (blue) and unmapped reads (red) in the K562 cell line are shown in figure (a). (PDF, 281 KB)
- Figure S4 - Length distribution of annotated introns in the gencode V7 annotation (PDF, 107 KB)
- Figure S5 - Boxplots for length distribution of mapped (blue) and unmapped reads (red) in the HelaS3 cell line (a). (PDF, 127 KB)
- Figure S6 - 454-read mappings in the HelaS3 cell-line (PDF, 132 KB)
- Figure S7 - Distribution of intron numbers per read for aligned 454-reads for the HelaS3 cell line (a). (PDF, 134 KB)
- Figure S8 - Four examples of 454-alignments whose intron-structures were not recapitulated by the short-read cufflinks predictions (PDF, 3 MB)
- Figure S9 - Example of an exon and its inclusion reads and exclusion reads in the K562 and HelaS3 cell-line (PDF, 230 KB)
