# Supplementary material for: Accurate Identification and Analysis of Human mRNA Isoforms Using Deep Long Read Sequencing
Source: G3 (Bethesda). 2013 Mar 1;3(3):387–97. doi: 10.1534/g3.112.004812 (PMC3583448; doi:10.1534/g3.112.004812)
Supplement: Supporting Information [file supp_3.3.387_004812SI.pdf]

G3: Genes | Genomes | Genetics

Accurate identification and analysis of human mRNA-isoforms using deep long read sequencing.

Hagen Tilgner[1], Debasish Raha[2], Lukas Habegger[3], Mohammed Mohiuddin[4], Mark Gerstein[3] and Michael Snyder[1]

1) Department of Genetics, Stanford University, Stanford CA, 94305

2) Department of Molecular, Cellular and Developmental Biology, Yale University, New Haven CT 06120

3) Program in Computational Biology and Department of Molecular Biophysics and Biochemistry, Yale University, New Haven CT 06120

4) Roche, 15 Commercial Street, Branford CT 06405

DOI: 10.1534/g3.112.004812

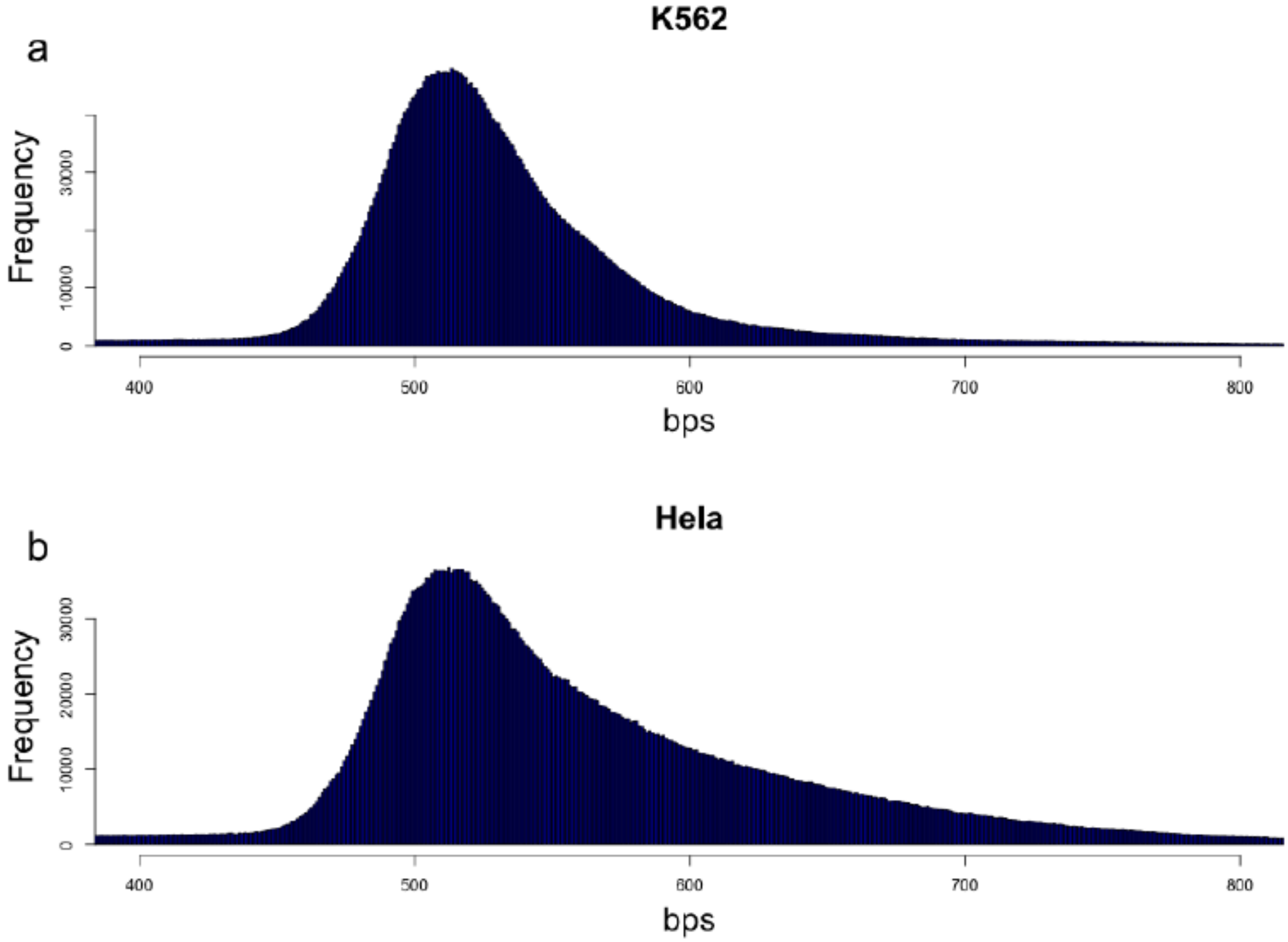

Figure S1: Read length distribution for reads in the K562 cell-line (a) and in the HelaS3 cell-line (b).

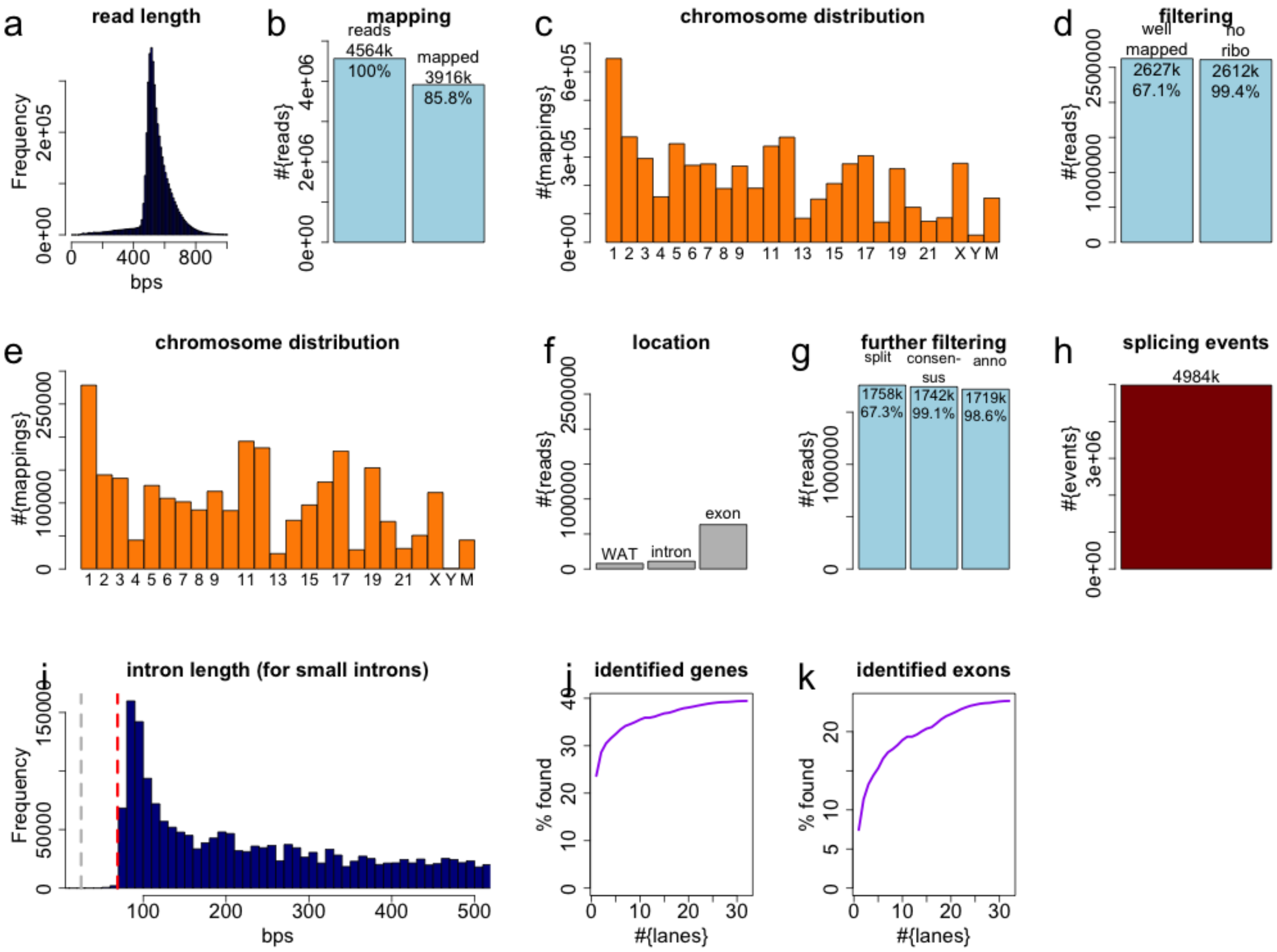

Figure S2: Read length histogram for the HelaS3 cell-line **(a)**. Total number of reads in the HelaS3 cell-line and number (and percentage) of reads that could be mapped using GMAP. Percentages in light blue bars are given with respect to the previous light blue bar **(b)**. Chromosome distribution of read-mappings **(c)**. Number of reads (and percentage) that were considered mapped with high confidence (well-mapped) and number of reads (and percentage) of reads that did not overlap ribosomal RNA genes **(d)**. Chromosome distribution of high confidence read mappings that did not overlap ribosomal RNA genes **(e)**. Number of reads falling entirely into intergenic, intronic and exonic regions **(f)**. Number and percentage (with respect to the previous light blue bar) of reads containing a split (first bar); number and percentage of reads containing at least one split and having intron-consensus di-nucleotides at the ends of all splits (second bar); number and percentage of reads containing at least one split and having intron-consensus di-nucleotides at the ends of all splits and having at least one split-end as an annotated splice site for all splits (third bar, **g**); Number of introns in these reads (with respect to last blue bar in previous figure, **h**). Intron length distribution for the previous introns, showing only introns of up to 500bps **(i)**. Percentage of annotated genes identified when using increasing number of reads **(j)**. Percentage of annotated exons identified when using increasing number of reads **(k)**.

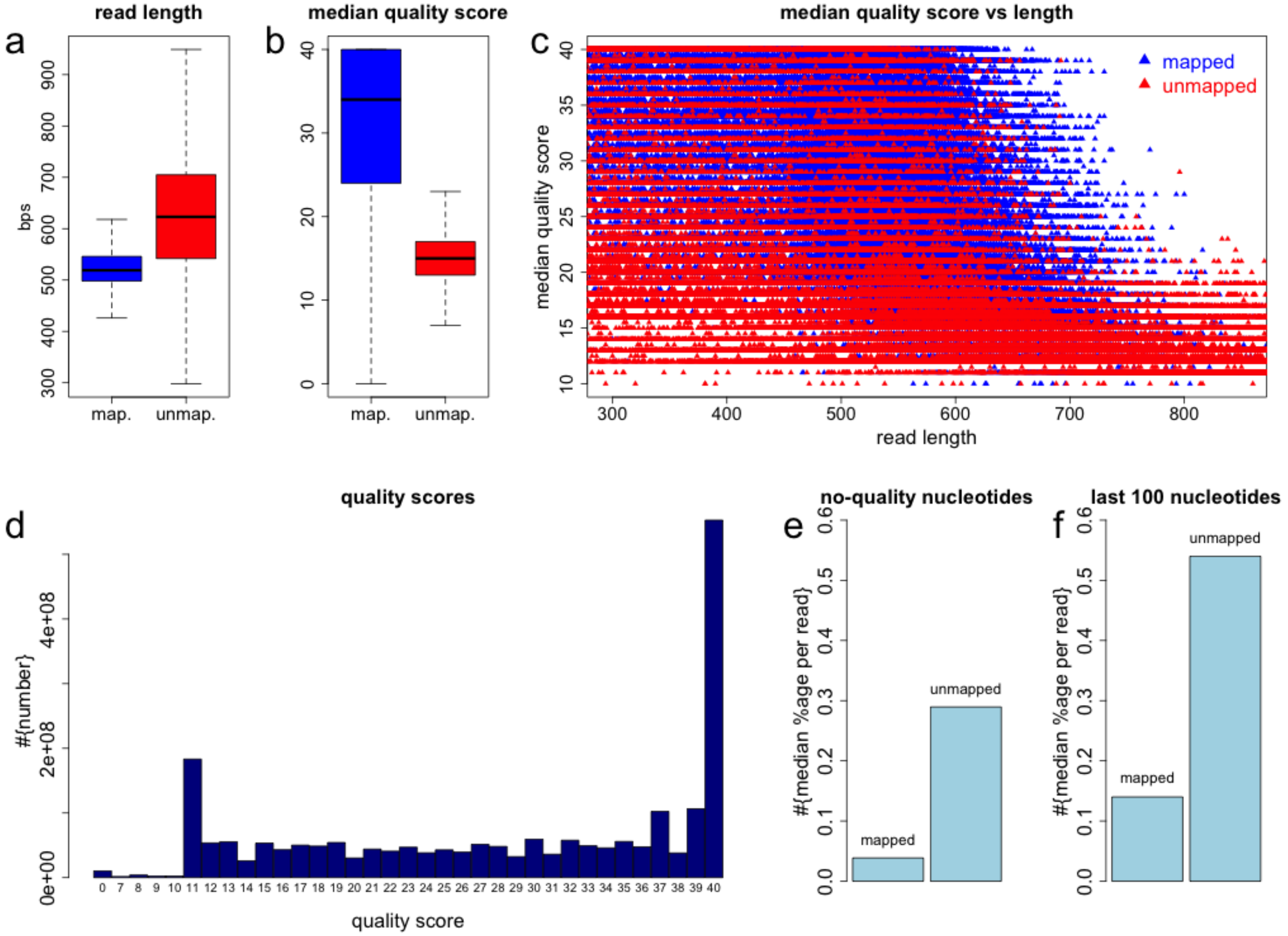

Figure S3: Boxplots for length distribution of mapped (blue) and unmapped reads (red) in the K562 cell line are shown in figure **(a)**. Surprisingly unmapped reads tended to be longer (median 623bps) than mapped reads (median length 519bps). We then calculated for each read the median of all bp-wise quality scores of the read. Boxplots for median-quality-score-distribution of mapped (blue) and unmapped reads (red) are shown in **(b)**. As expected unmapped reads had much lower qualities than mapped reads. A scatterplot of both variables **(c)** shows that longer read length and lower scores tend to co-occur for unmapped reads. The histogram of all bp-wise quality scores of all reads revealed a strong peak for quality-score 11 with essentially no lower quality scores suggesting that this bin is enriched in random or no-quality base-calls **(d)**. The increased read length of unmapped reads appears to be mainly due to large numbers of very low quality nucleotides **(e)**. When counting the number of nucleotides with a quality value of 11 or less, we found unmapped reads to show a median of 179 (a median of 28.9% of the these reads) such nucleotides. Mapped reads on the other hand showed a median of 19 (a median of 3.9% of the nucleotides of these reads) such nucleotides. When limiting this analysis to the last 100bps of unmapped and mapped reads, we found a median of 54 no-quality nucleotides (quality score$\leq$11) for unmapped reads and 14 for mapped reads **(f)**. Hence, the nucleotides that make unmapped reads longer than mapped reads are extremely enriched for no-quality nucleotides, although unmapped reads also harbor no-quality nucleotides in more 5 prime sequences. Generally, all reads harbor larger numbers of no-quality nucleotides towards the end of the read.

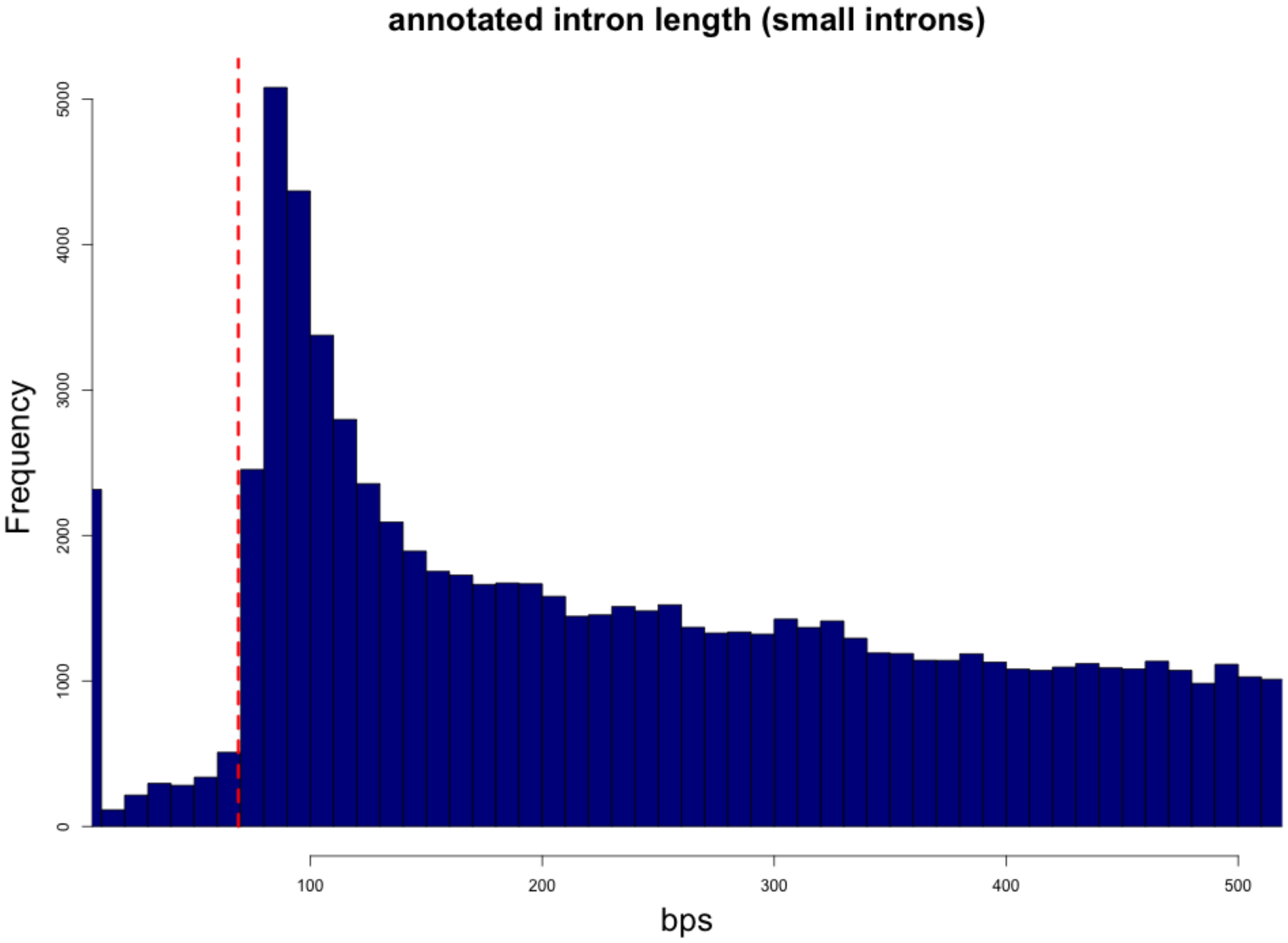

Figure S4: Length distribution of annotated introns in the gencode V7 annotation.

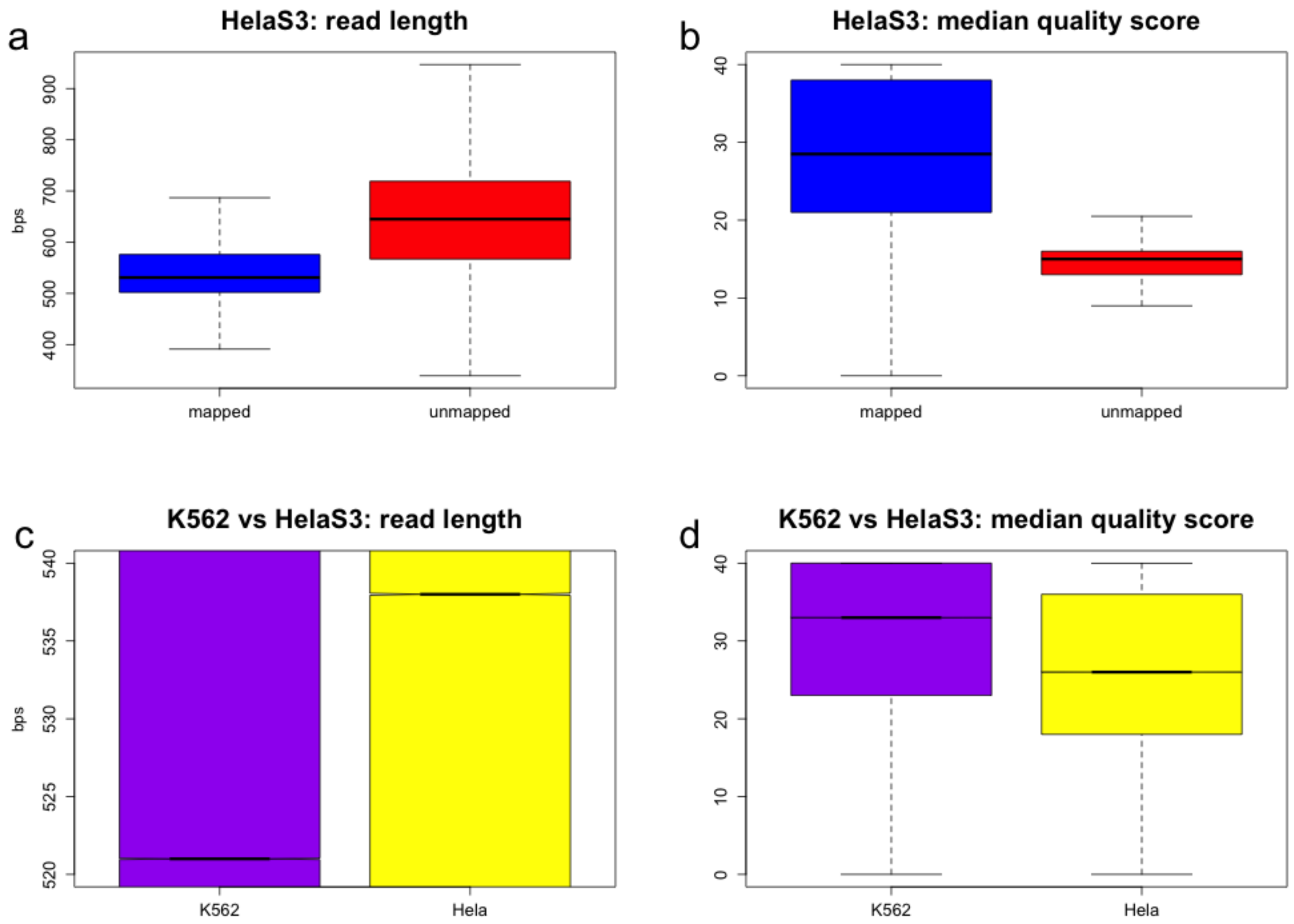

Figure S5: Boxplots for length distribution of mapped (blue) and unmapped reads (red) in the HelaS3 cell line **(a)**. Boxplots for median-quality-score-distribution of mapped (blue) and unmapped reads. For each read we calculated the median of all bp-wise quality scores **(b)**. Read-length-boxplots for all reads in the K562 cell line and in the HelaS3 cell-line **(c)**. Median-quality-score boxplots for all reads in the K562 cell line and in the HelaS3 cell-line **(d)**.

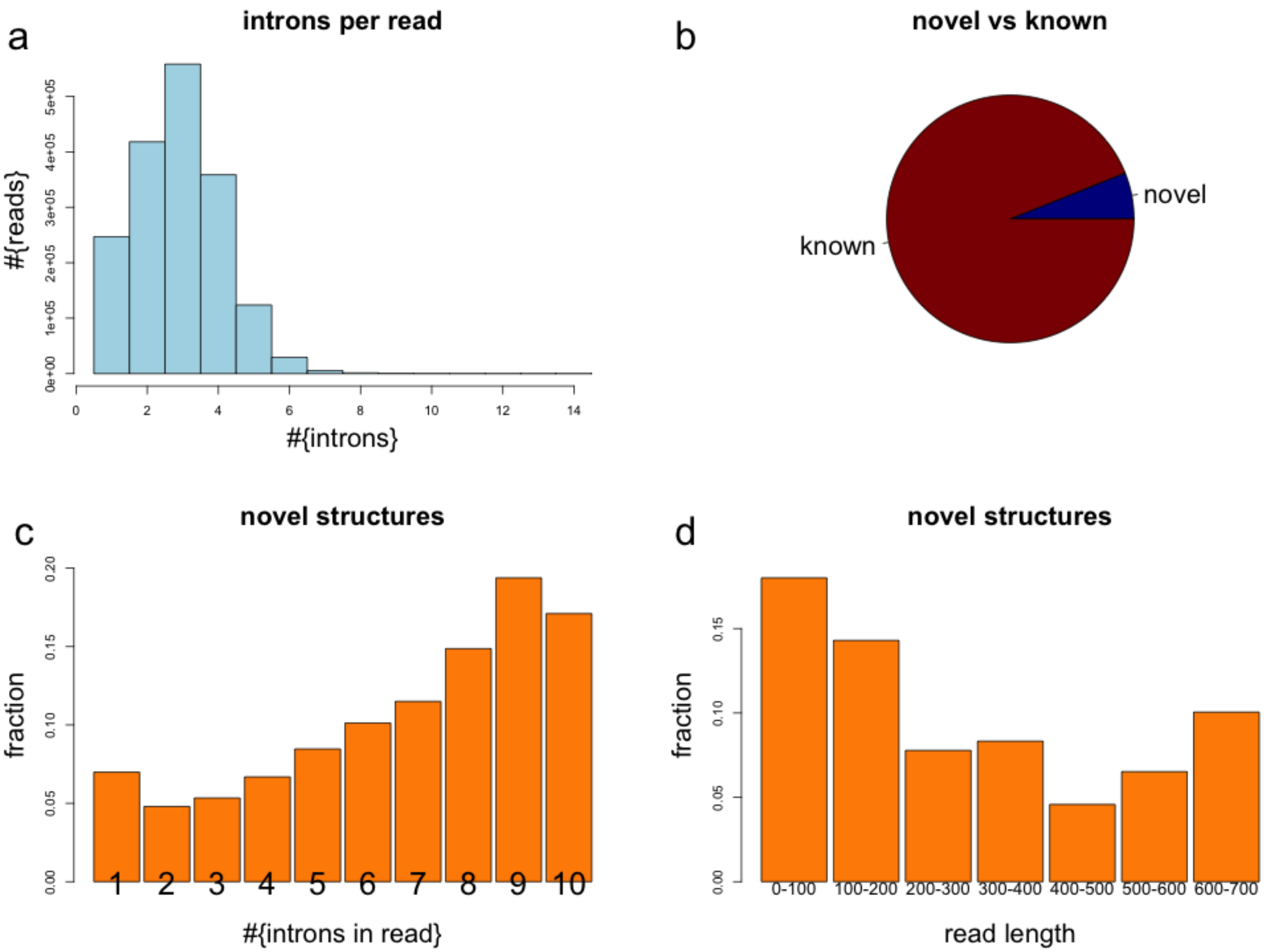

Figure S6: 454-read mappings in the HelaS3 cell-line. Distribution of intron number in aligned reads (with consensus splits, **a**). Pie chart of partial 454 gene structures that (i) correspond to parts of annotated gene structures and (ii) those that do not correspond to parts of annotated gene structures **(b)**. Fraction of reads that are not included in annotated gene structures as a function of intron number in the read-alignments **(c)**. Fraction of reads that are not included in annotated gene structures as a function of read-length. Note that there are very few reads that have between 0-400bps **(d)**.

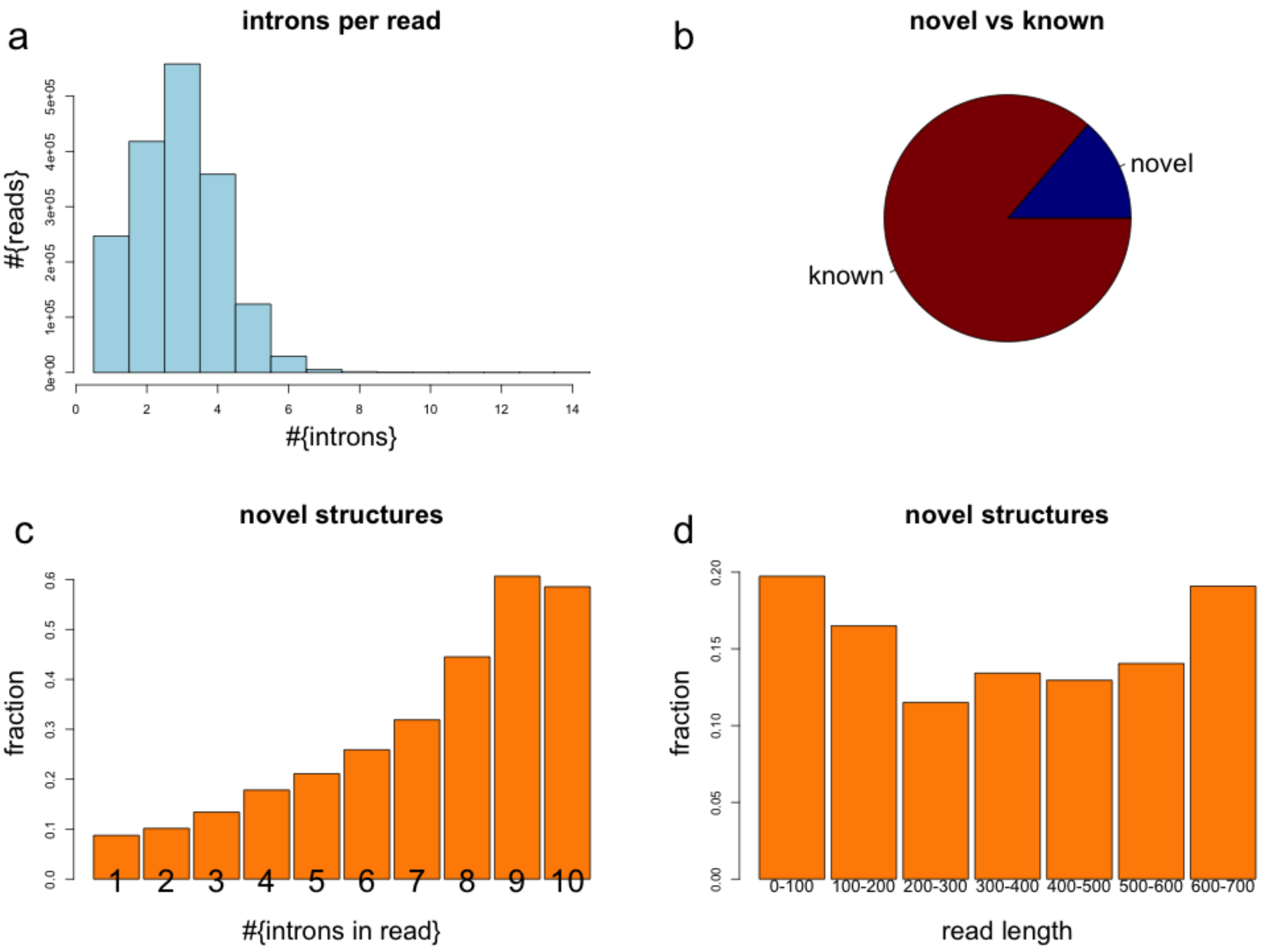

Figure S7: Distribution of intron numbers per read for aligned 454-reads for the HelaS3 cell line **(a)**. Pie chart (for the HelaS3 cell line) of partial 454 gene structures that (i) correspond to parts of full-length transcript structures predicted using ENCODE short reads and (ii) those that do not correspond to parts of these predicted transcript structures **(b)**. Fraction of reads that are not included in predicted transcript structures (based on ENCODE short reads) as a function of intron number in the read-alignments **(c)**. Fraction of reads that are not included in predicted transcript structures as a function of read-length. Note that there are very few reads that have between 0-400bps **(d)**.

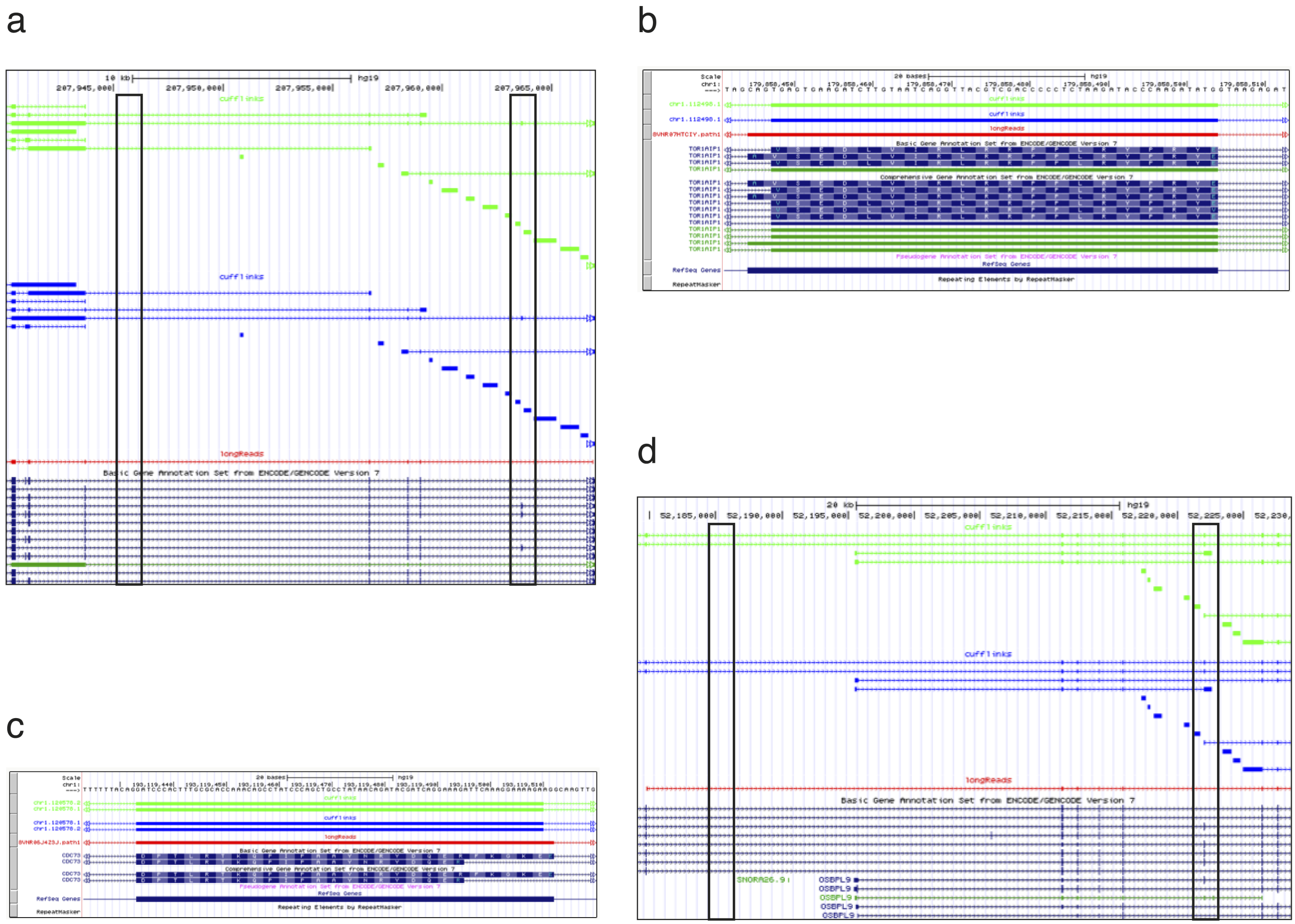

Figure S8: Four examples of 454-alignments whose intron-structures were not recapitulated by the short-read cufflinks predictions. In these plots, we show only one 454-alignment (red, the one giving an intron-structure that is not recapitulated by the short-read cufflinks approach). For short-read-cufflinks-transcripts, we show all original transcripts (green), and those in which we removed very small introns (<25bp, blue). An example in which an exon-skipping event (black box to the right) occurs within the same molecule with a 5 prime extended RNA molecule, a structure in-line with the annotation (black box to the left). The short read-cufflinks approach predicts both events, but not within the same transcript **(a)**. A case of an alternative acceptor. Note, that in this case, both the short-read-cufflinks transcript as well as the 454-alignment are consistent with annotated transcripts. Since all short-read-cufflinks transcripts are shown, these transcripts do not give all the information, that 454-alignments provide **(b)**. A case of an alternative donor, in which the short-read-cufflinks transcript does not correspond to the annotation **(c)**. A case similar to the case shown in subfigure a **(d)**.

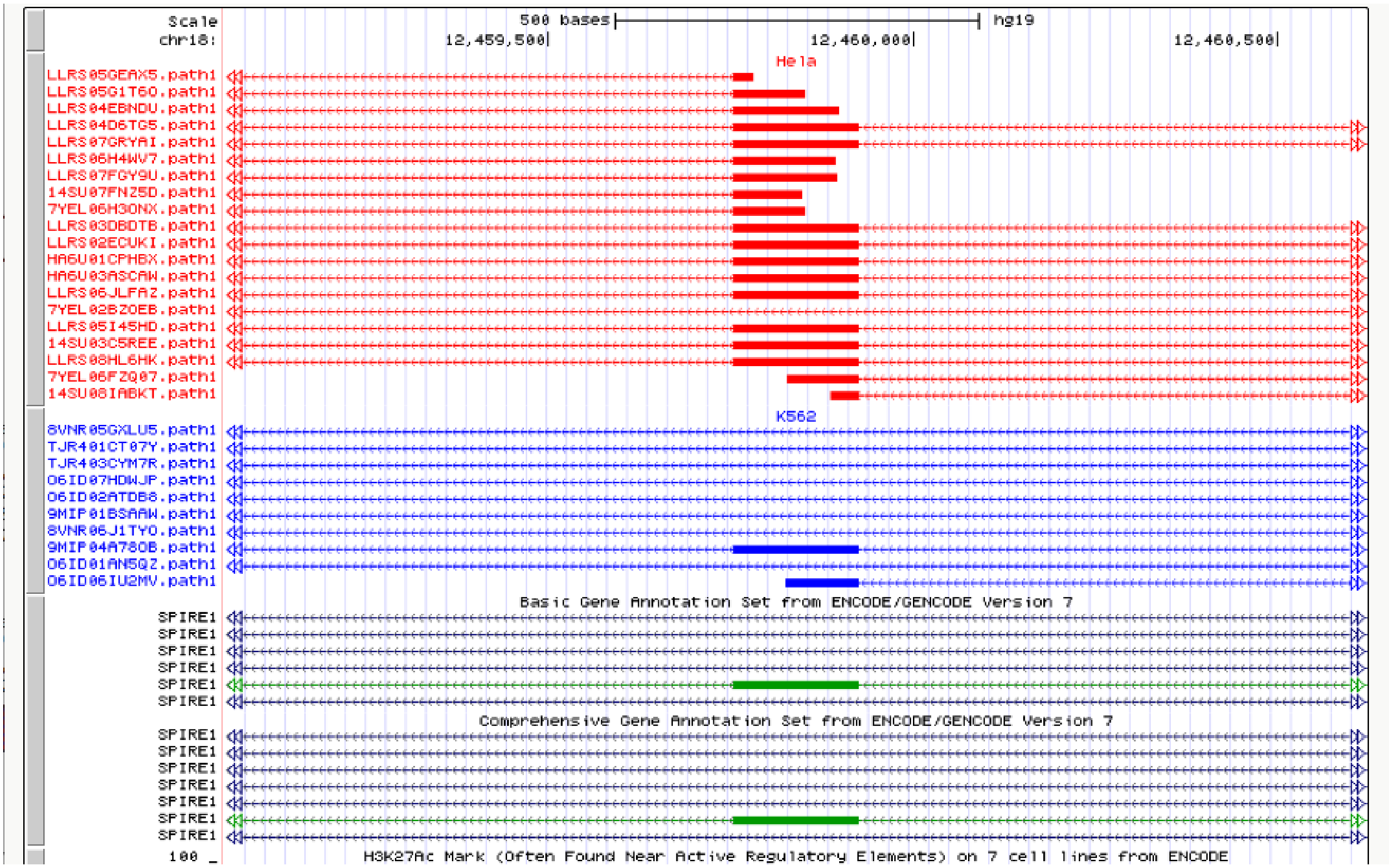

Figure S9: : Example of an exon and its inclusion reads and exclusion reads in the K562 and HelaS3 cell line. This example suggests higher exon inclusion in the HelaS3 cell line than in the K562 cell-line.
