# Supplementary figures and images for: Accurate Identification and Analysis of Human mRNA Isoforms Using Deep Long Read Sequencing
Source: G3 (Bethesda). 2013 Mar 1;3(3):387–97. doi: 10.1534/g3.112.004812 (PMC3583448; doi:10.1534/g3.112.004812)

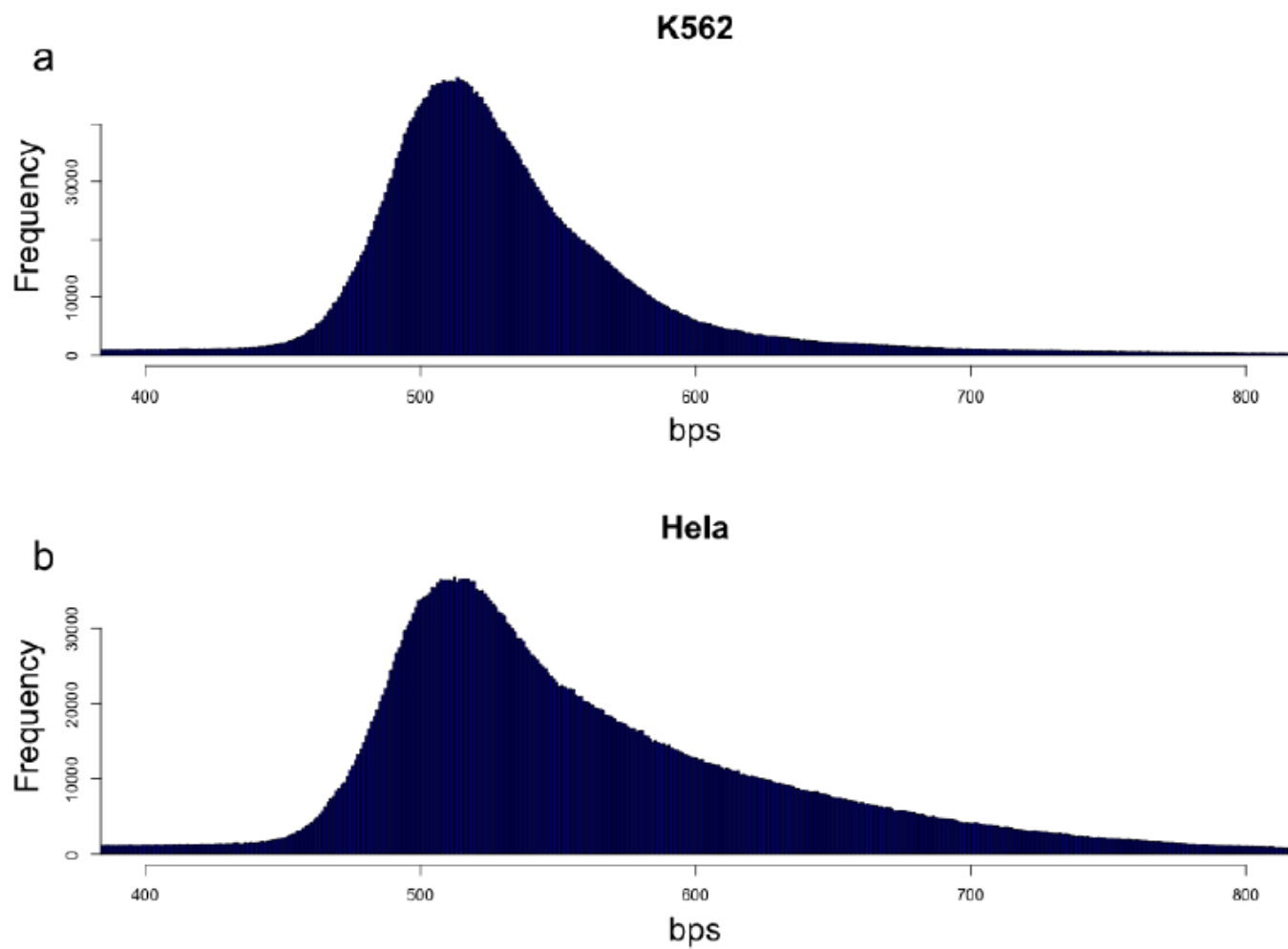

**Figure S1:** Read length distribution for reads in the K562 cell-line (a) and in the HeLaS3 cell-line (b).

Supplement: Supporting Information [file supp_3.3.387_FigureS1.pdf]

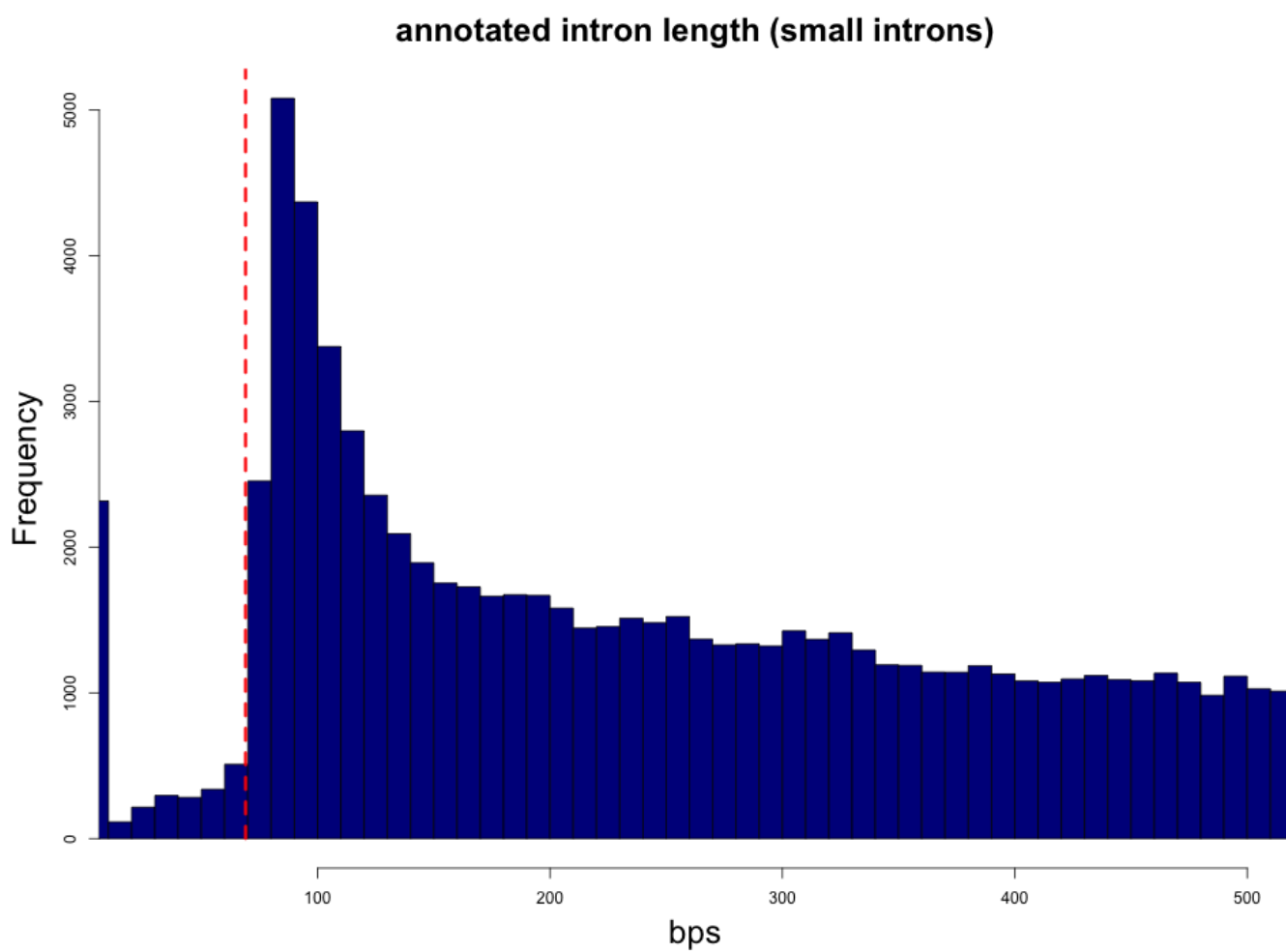

**Figure S4:** Length distribution of annotated introns in the gencode V7 annotation.

Supplement: Supporting Information [file supp_3.3.387_FigureS4.pdf]
