# Supplementary material for: Accurate Identification and Analysis of Human mRNA Isoforms Using Deep Long Read Sequencing
Source: G3 (Bethesda). 2013 Mar 1;3(3):387–97. doi: 10.1534/g3.112.004812 (PMC3583448; doi:10.1534/g3.112.004812)
Supplement: Supporting Information [file supp_3.3.387_FigureS2.pdf]

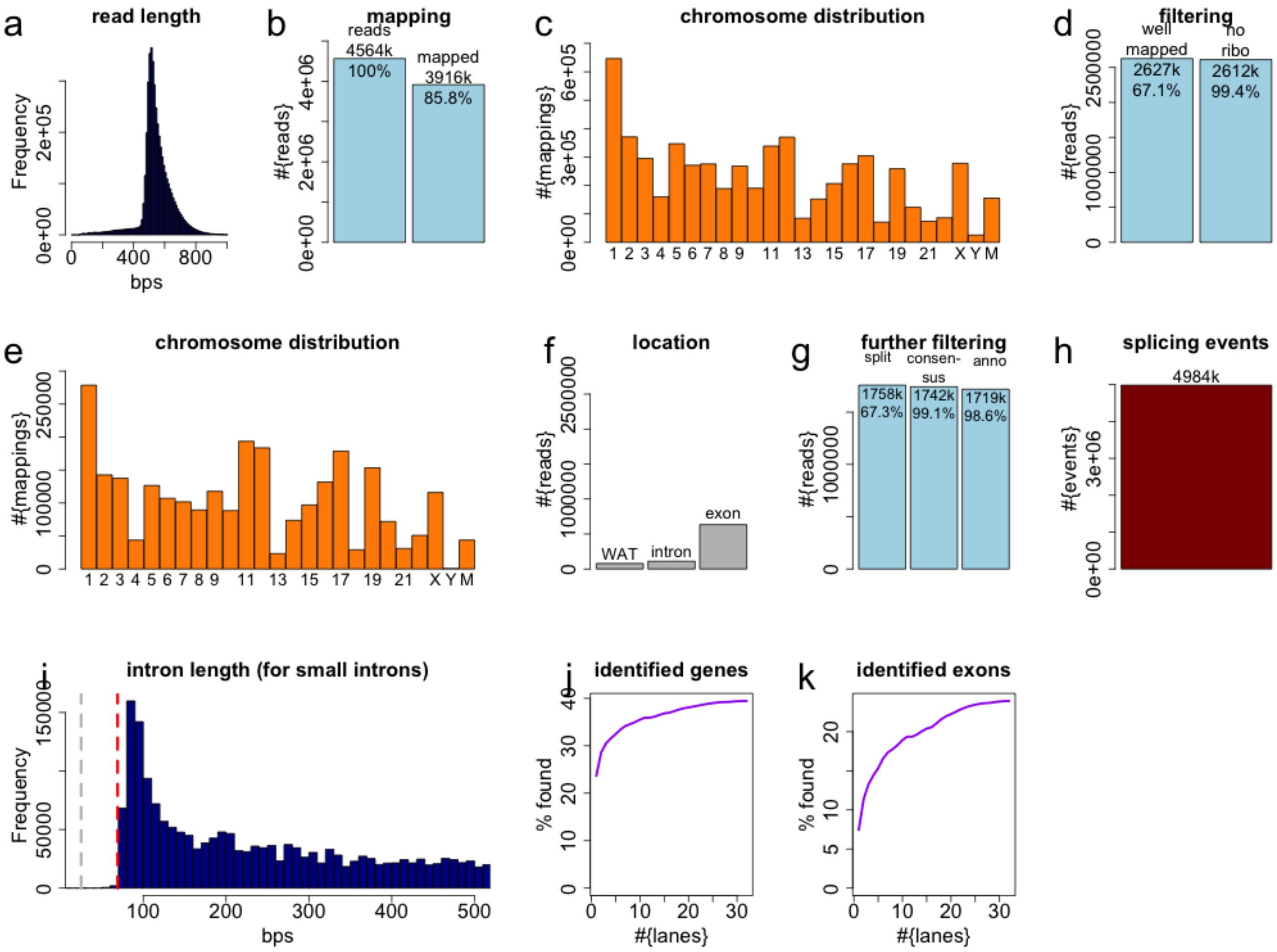

Figure S2: Read length histogram for the HelaS3 cell-line **(a)**. Total number of reads in the HelaS3 cell-line and number (and percentage) of reads that could be mapped using GMAP. Percentages in light blue bars are given with respect to the previous light blue bar **(b)**. Chromosome distribution of read-mappings **(c)**. Number of reads (and percentage) that were considered mapped with high confidence (well-mapped) and number of reads (and percentage) of reads that did not overlap ribosomal RNA genes **(d)**. Chromosome distribution of high confidence read mappings that did not overlap ribosomal RNA genes **(e)**. Number of reads falling entirely into intergenic, intronic and exonic regions **(f)**. Number and percentage (with respect to the previous light blue bar) of reads containing a split (first bar); number and percentage of reads containing at least one split and having intron-consensus di-nucleotides at the ends of all splits (second bar); number and percentage of reads containing at least one split and having intron-consensus di-nucleotides at the ends of all splits and having at least one split-end as an annotated splice site for all splits (third bar, **g**); Number of introns in these reads (with respect to last blue bar in previous figure, **h**). Intron length distribution for the previous introns, showing only introns of up to 500bps **(i)**. Percentage of annotated genes identified when using increasing number of reads **(j)**. Percentage of annotated exons identified when using increasing number of reads **(k)**.
