# Supplementary material for: Accurate Identification and Analysis of Human mRNA Isoforms Using Deep Long Read Sequencing
Source: G3 (Bethesda). 2013 Mar 1;3(3):387–97. doi: 10.1534/g3.112.004812 (PMC3583448; doi:10.1534/g3.112.004812)
Supplement: Supporting Information [file supp_3.3.387_FigureS3.pdf]

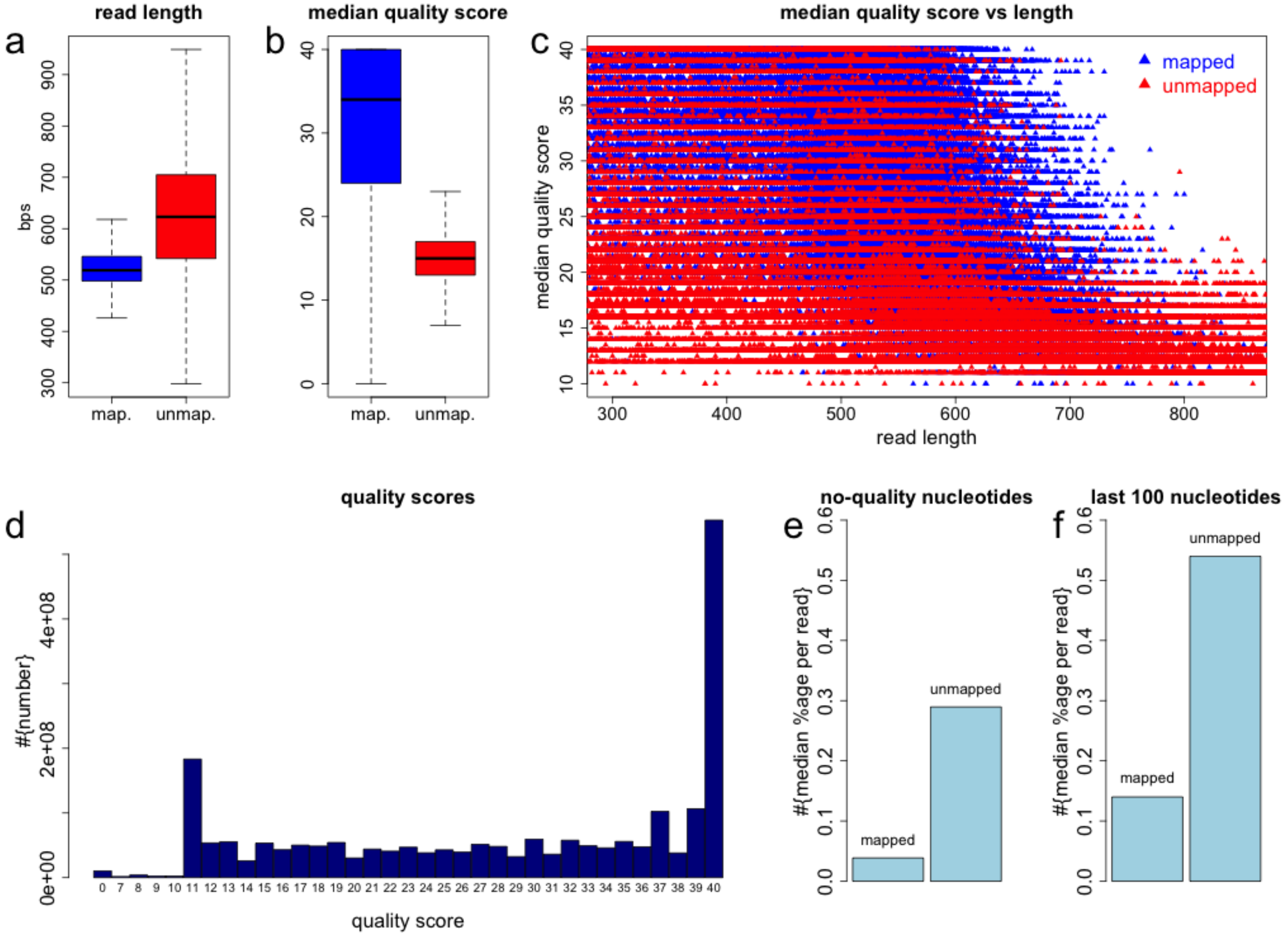

Figure S3: Boxplots for length distribution of mapped (blue) and unmapped reads (red) in the K562 cell line are shown in figure **(a)**. Surprisingly unmapped reads tended to be longer (median 623bps) than mapped reads (median length 519bps). We then calculated for each read the median of all bp-wise quality scores of the read. Boxplots for median-quality-score-distribution of mapped (blue) and unmapped reads (red) are shown in **(b)**. As expected unmapped reads had much lower qualities than mapped reads. A scatterplot of both variables **(c)** shows that longer read length and lower scores tend to co-occur for unmapped reads. The histogram of all bp-wise quality scores of all reads revealed a strong peak for quality-score 11 with essentially no lower quality scores suggesting that this bin is enriched in random or no-quality base-calls **(d)**. The increased read length of unmapped reads appears to be mainly due to large numbers of very low quality nucleotides **(e)**. When counting the number of nucleotides with a quality value of 11 or less, we found unmapped reads to show a median of 179 (a median of 28.9% of the these reads) such nucleotides. Mapped reads on the other hand showed a median of 19 (a median of 3.9% of the nucleotides of these reads) such nucleotides. When limiting this analysis to the last 100bps of unmapped and mapped reads, we found a median of 54 no-quality nucleotides (quality score$\leq$11) for unmapped reads and 14 for mapped reads **(f)**. Hence, the nucleotides that make unmapped reads longer than mapped reads are extremely enriched for no-quality nucleotides, although unmapped reads also harbor no-quality nucleotides in more 5 prime sequences. Generally, all reads harbor larger numbers of no-quality nucleotides towards the end of the read.
