# Supplementary material for: Accurate Identification and Analysis of Human mRNA Isoforms Using Deep Long Read Sequencing
Source: G3 (Bethesda). 2013 Mar 1;3(3):387–97. doi: 10.1534/g3.112.004812 (PMC3583448; doi:10.1534/g3.112.004812)
Supplement: Supporting Information [file supp_3.3.387_FigureS5.pdf]

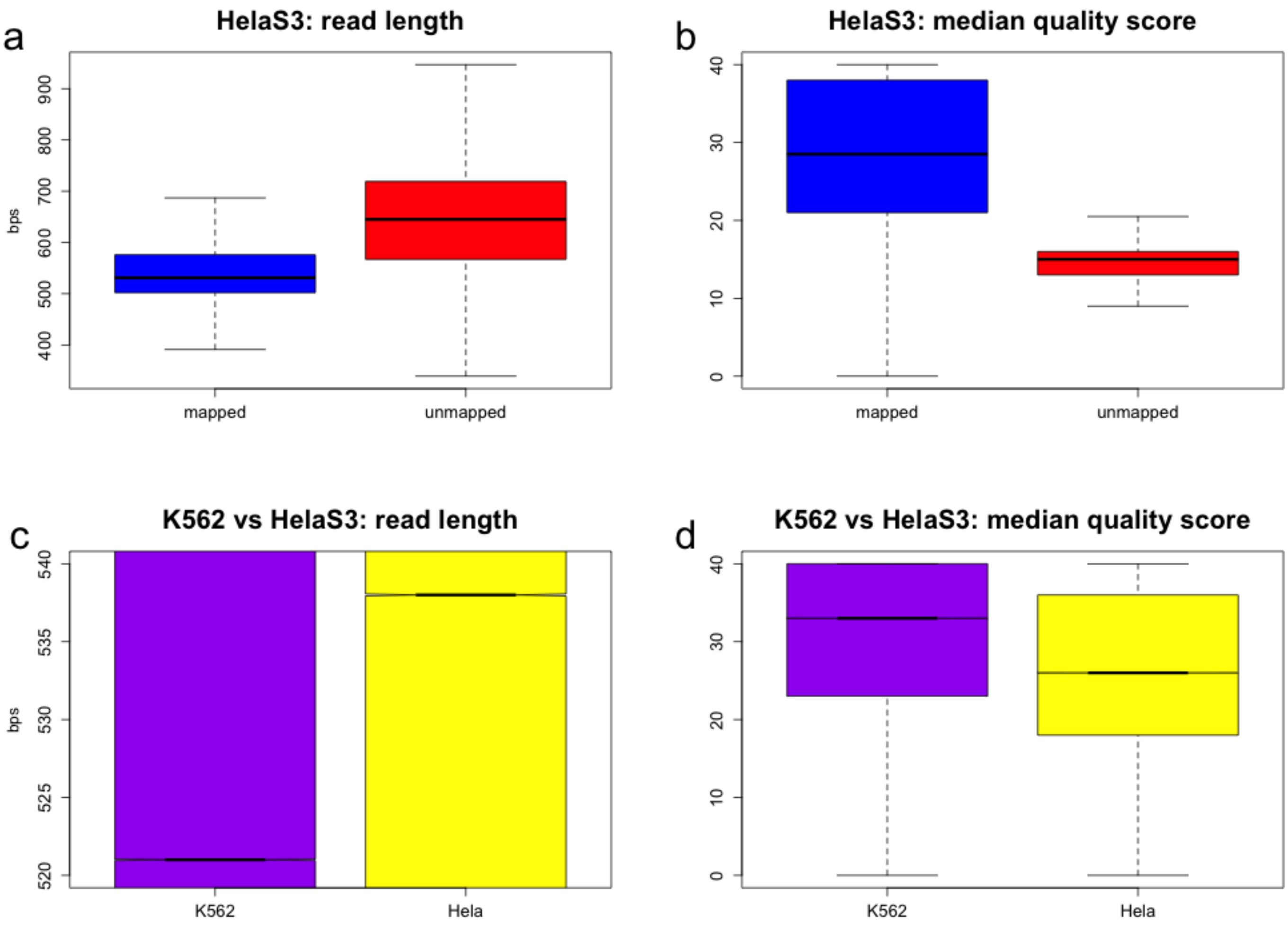

Figure S5: Boxplots for length distribution of mapped (blue) and unmapped reads (red) in the HelaS3 cell line **(a)**. Boxplots for median-quality-score-distribution of mapped (blue) and unmapped reads. For each read we calculated the median of all bp-wise quality scores **(b)**. Read-length-boxplots for all reads in the K562 cell line and in the HelaS3 cell-line **(c)**. Median-quality-score boxplots for all reads in the K562 cell line and in the HelaS3 cell-line **(d)**.
