# Supplementary material for: Accurate Identification and Analysis of Human mRNA Isoforms Using Deep Long Read Sequencing
Source: G3 (Bethesda). 2013 Mar 1;3(3):387–97. doi: 10.1534/g3.112.004812 (PMC3583448; doi:10.1534/g3.112.004812)
Supplement: Supporting Information [file supp_3.3.387_FigureS6.pdf]

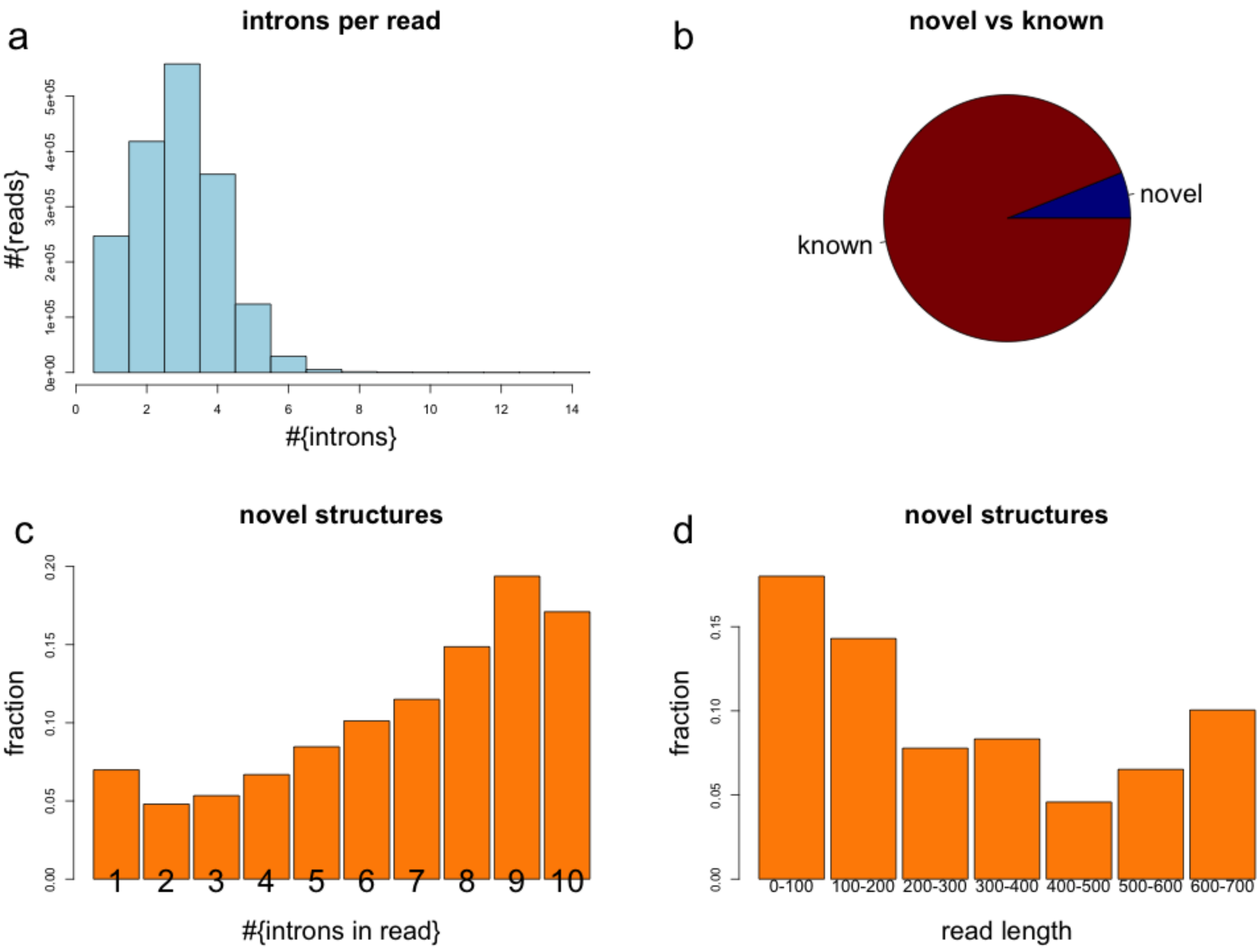

Figure S6: 454-read mappings in the HelaS3 cell-line. Distribution of intron number in aligned reads (with consensus splits, **a**). Pie chart of partial 454 gene structures that (i) correspond to parts of annotated gene structures and (ii) those that do not correspond to parts of annotated gene structures **(b)**. Fraction of reads that are not included in annotated gene structures as a function of intron number in the read-alignments **(c)**. Fraction of reads that are not included in annotated gene structures as a function of read-length. Note that there are very few reads that have between 0-400bps **(d)**.
