# Supplementary material for: Accurate Identification and Analysis of Human mRNA Isoforms Using Deep Long Read Sequencing
Source: G3 (Bethesda). 2013 Mar 1;3(3):387–97. doi: 10.1534/g3.112.004812 (PMC3583448; doi:10.1534/g3.112.004812)
Supplement: Supporting Information [file supp_3.3.387_FigureS7.pdf]

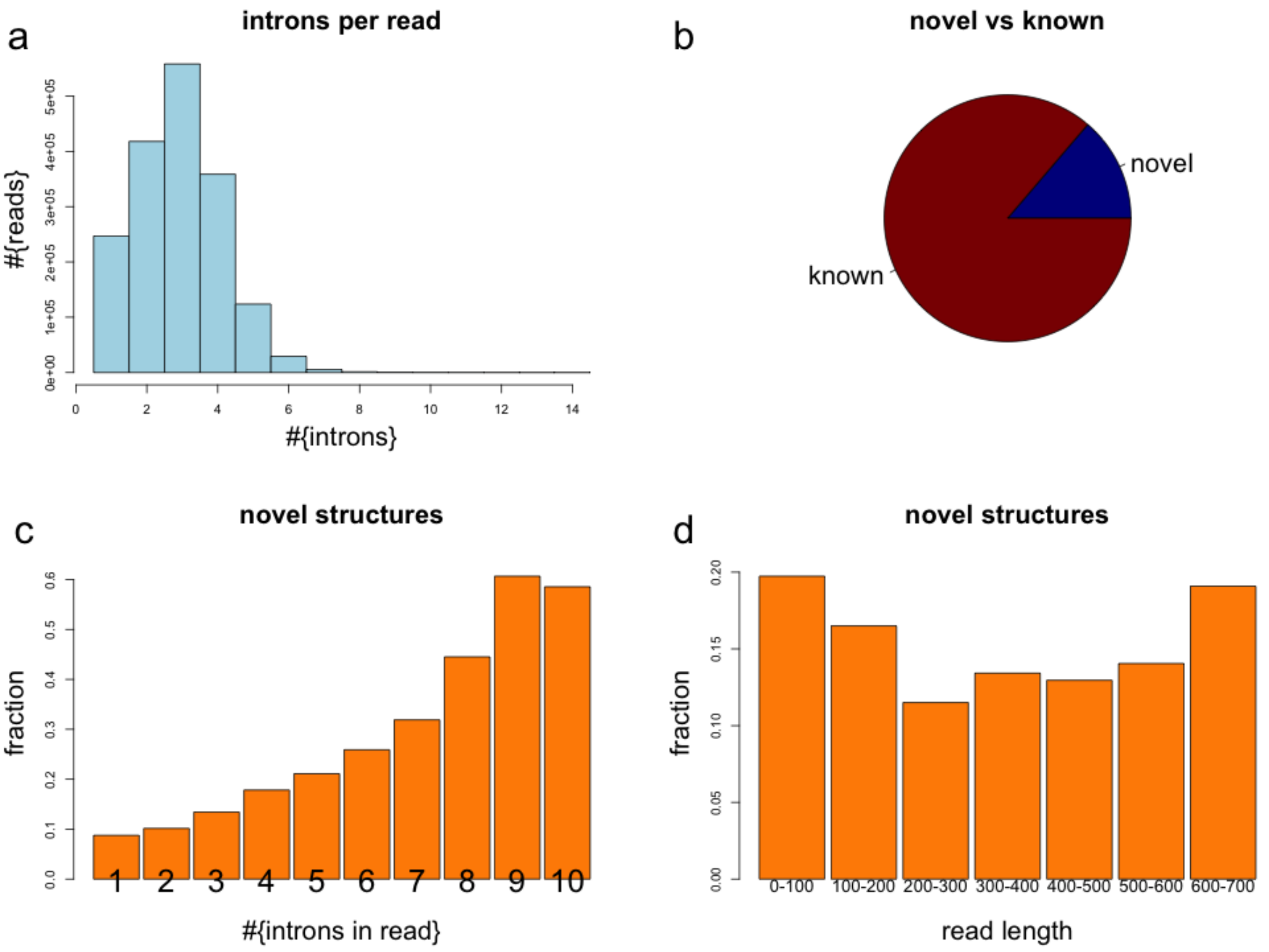

Figure S7: Distribution of intron numbers per read for aligned 454-reads for the HelaS3 cell line **(a)**. Pie chart (for the HelaS3 cell line) of partial 454 gene structures that (i) correspond to parts of full-length transcript structures predicted using ENCODE short reads and (ii) those that do not correspond to parts of these predicted transcript structures **(b)**. Fraction of reads that are not included in predicted transcript structures (based on ENCODE short reads) as a function of intron number in the read-alignments **(c)**. Fraction of reads that are not included in predicted transcript structures as a function of read-length. Note that there are very few reads that have between 0-400bps **(d)**.
