# Supplementary material for: Accurate Identification and Analysis of Human mRNA Isoforms Using Deep Long Read Sequencing
Source: G3 (Bethesda). 2013 Mar 1;3(3):387–97. doi: 10.1534/g3.112.004812 (PMC3583448; doi:10.1534/g3.112.004812)
Supplement: Supporting Information [file supp_3.3.387_FigureS8.pdf]

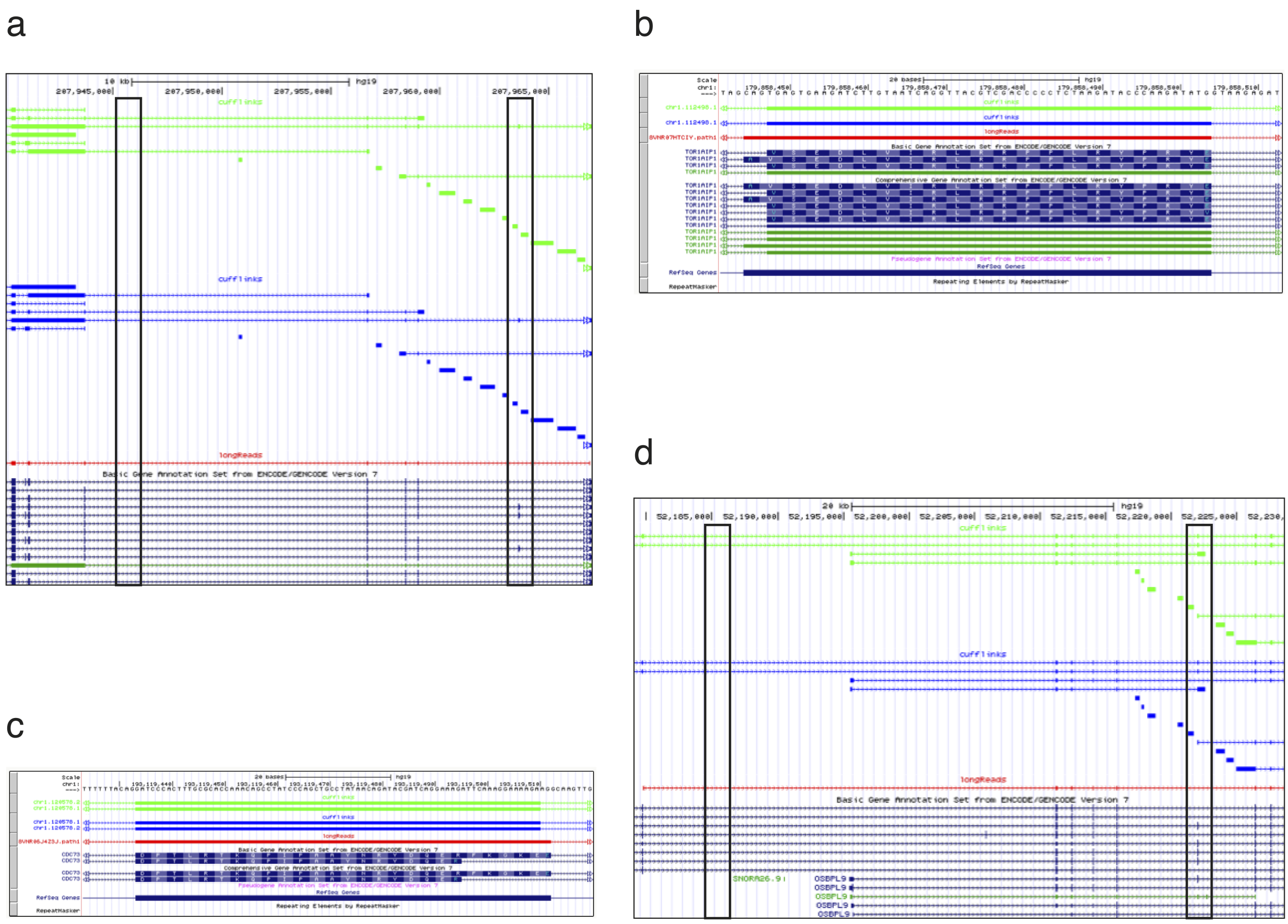

Figure S8: Four examples of 454-alignments whose intron-structures were not recapitulated by the short-read cufflinks predictions. In these plots, we show only one 454-alignment (red, the one giving an intron-structure that is not recapitulated by the short-read cufflinks approach). For short-read-cufflinks-transcripts, we show all original transcripts (green), and those in which we removed very small introns (<25bp, blue). An example in which an exon-skipping event (black box to the right) occurs within the same molecule with a 5 prime extended RNA molecule, a structure in-line with the annotation (black box to the left). The short read-cufflinks approach predicts both events, but not within the same transcript **(a)**. A case of an alternative acceptor. Note, that in this case, both the short-read-cufflinks transcript as well as the 454-alignment are consistent with annotated transcripts. Since all short-read-cufflinks transcripts are shown, these transcripts do not give all the information, that 454-alignments provide **(b)**. A case of an alternative donor, in which the short-read-cufflinks transcript does not correspond to the annotation **(c)**. A case similar to the case shown in subfigure a **(d)**.
