# Supplementary material for: Accurate Identification and Analysis of Human mRNA Isoforms Using Deep Long Read Sequencing
Source: G3 (Bethesda). 2013 Mar 1;3(3):387–97. doi: 10.1534/g3.112.004812 (PMC3583448; doi:10.1534/g3.112.004812)
Supplement: Supporting Information [file supp_3.3.387_FigureS9.pdf]

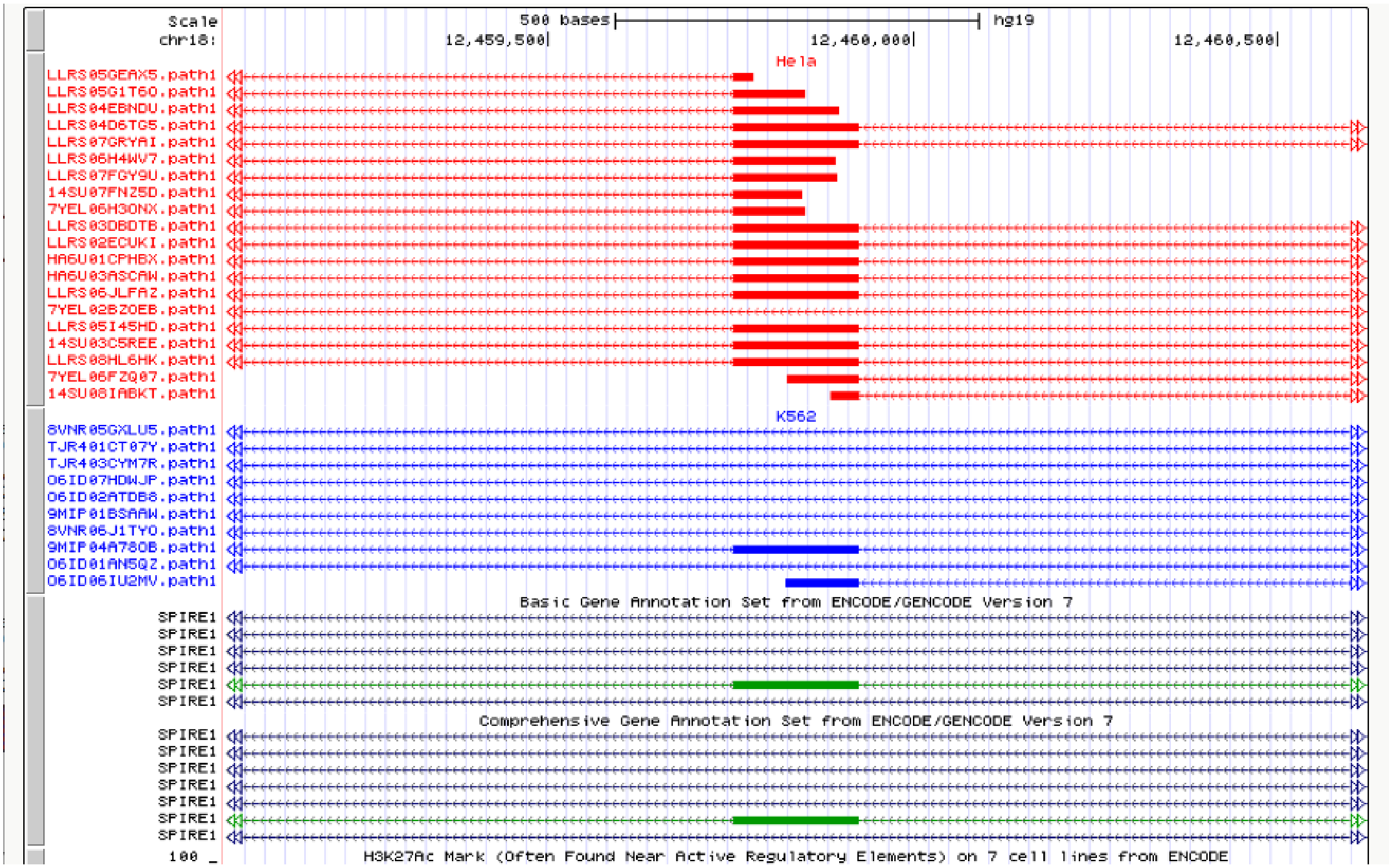

Figure S9: : Example of an exon and its inclusion reads and exclusion reads in the K562 and HelaS3 cell line. This example suggests higher exon inclusion in the HelaS3 cell line than in the K562 cell-line.
